# Human Papillomavirus 42 Drives Digital Papillary Adenocarcinoma and Elicits a Germ Cell–like Program Conserved in HPV-Positive Cancers

**DOI:** 10.1158/2159-8290.CD-22-0489

**Published:** 2022-10-10

**Authors:** Lukas Leiendecker, Tobias Neumann, Pauline S. Jung, Shona M. Cronin, Thomas L. Steinacker, Alexander Schleiffer, Michael Schutzbier, Karl Mechtler, Thibault Kervarrec, Estelle Laurent, Kamel Bachiri, Etienne Coyaud, Rajmohan Murali, Klaus J. Busam, Babak Itzinger-Monshi, Reinhard Kirnbauer, Lorenzo Cerroni, Eduardo Calonje, Arno Rütten, Frank Stubenrauch, Klaus G. Griewank, Thomas Wiesner, Anna C. Obenauf

**Affiliations:** 1 Research Institute of Molecular Pathology (IMP), Vienna BioCenter (VBC), Vienna, Austria.; 2 Vienna BioCenter PhD Program, Doctoral School of the University at Vienna and Medical University of Vienna, Vienna BioCenter (VBC), Vienna, Austria.; 3 Quantro Therapeutics, Vienna, Austria.; 4 Department of Dermatology, Medical University of Vienna, Vienna, Austria.; 5 Institute of Molecular Biotechnology (IMBA), Vienna BioCenter (VBC), Vienna, Austria.; 6 The Gregor Mendel Institute of Molecular Plant Biology of the Austrian Academy of Sciences (GMI), Vienna BioCenter (VBC), Vienna, Austria.; 7 Department of Pathology, University Hospital Center of Tours, University of Tours, Tours, France.; 8 PRISM INSERM U1192, Université de Lille, Villeneuve d'Ascq, France.; 9 Department of Pathology and Laboratory Medicine, Memorial Sloan Kettering Cancer Center, New York, New York.; 10 Department of Dermatology, Hospital Rudolfstiftung, Vienna, Austria.; 11 Department of Dermatology, Medical University of Graz, Graz, Austria.; 12 Department of Dermatopathology, St John's Institute of Dermatology, St Thomas’ Hospital, London, United Kingdom.; 13 Dermatopathology Friedrichshafen, Friedrichshafen, Germany.; 14 University Hospital Tuebingen, Institute for Medical Virology and Epidemiology of Viral Diseases, Tuebingen, Germany.; 15 Department of Dermatology, University Hospital Essen, University of Duisburg, German Cancer Consortium (DKTK), Partner Site, Essen, Germany.; 16 Department of Pathology, Medical University of Vienna, Vienna, Austria.

## Abstract

The skin is exposed to viral pathogens, but whether they contribute to the oncogenesis of skin cancers has not been systematically explored. Here we investigated 19 skin tumor types by analyzing off-target reads from commonly available next-generation sequencing data for viral pathogens. We identified human papillomavirus 42 (HPV42) in 96% (*n* = 45/47) of digital papillary adenocarcinoma (DPA), an aggressive cancer occurring on the fingers and toes. We show that HPV42, so far considered a nononcogenic, “low-risk” HPV, recapitulates the molecular hallmarks of oncogenic, “high-risk” HPVs. Using machine learning, we find that HPV-driven transformation elicits a germ cell–like transcriptional program conserved throughout all HPV-driven cancers (DPA, cervical carcinoma, and head and neck cancer). We further show that this germ cell–like transcriptional program, even when reduced to the top two genes (*CDKN2A* and *SYCP2*), serves as a fingerprint of oncogenic HPVs with implications for early detection, diagnosis, and therapy of all HPV-driven cancers.

**Significance:**

We identify HPV42 as a uniform driver of DPA and add a new member to the short list of tumorigenic viruses in humans. We discover that all oncogenic HPVs evoke a germ cell–like transcriptional program with important implications for detecting, diagnosing, and treating all HPV-driven cancers.

*See related commentary by Starrett et al., p. 17.*

*This article is highlighted in the In This Issue feature, p. 1*

## Teaser

Human papillomavirus (HPV) 42 is a novel tumorigenic virus that drives a germ cell–like program conserved in all HPV-driven tumors that distinguishes HPV-driven from mutation-driven tumors and outperforms established clinical markers.

## INTRODUCTION

Up to 15% of human cancers are caused by microbial pathogens, most notably by viruses that interfere with signaling pathways maintaining cellular homeostasis (1). Prominent examples include cervical carcinoma and head and neck cancer caused by oncogenic human papillomavirus (HPV), Merkel cell carcinoma by Merkel cell polyomavirus (MCV), Kaposi sarcoma by human herpesvirus 8 (HHV8), or hepatocellular carcinoma associated with chronic hepatitis B/C virus infection (2). Many virus-associated cancers occur at the interface between the human body and the environment, such as the mucosal epithelium or skin (1), but the contribution of viral pathogens to skin cancers has not been systematically investigated.

## RESULTS

### HPV42 Is Associated with Digital Papillary Adenocarcinoma

**Figure 1.**
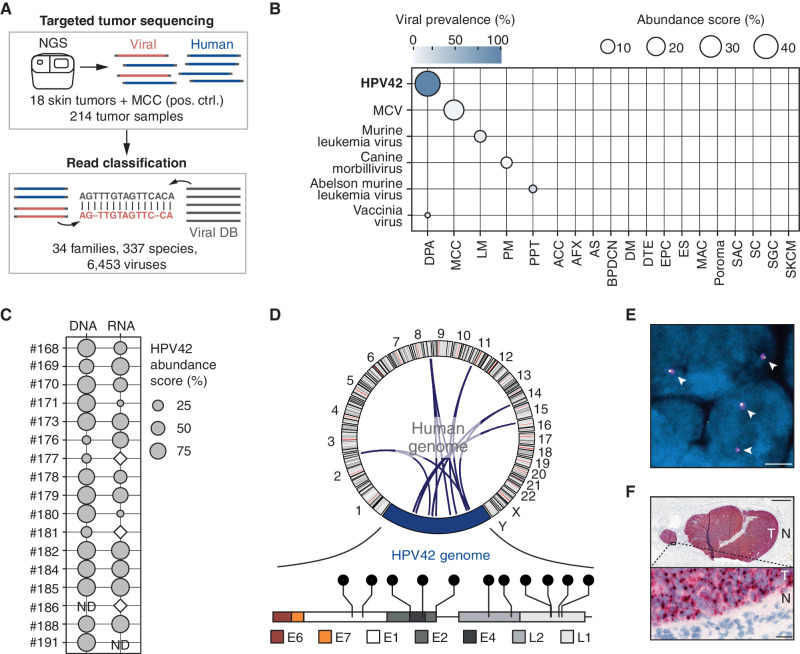
HPV42 is associated with DPA. **A,** Overview of unbiased screening approach for detection of vertebrate-infecting viruses in targeted next-generation sequencing (NGS) data. Targeted NGS data from 18 skin tumor types and MCC (positive control, pos. ctrl.) were analyzed with the centrifuge metagenomic classifier utilizing a custom viral database (Viral DB). **B,** Virus abundance score in targeted NGS data of 214 human tumor samples. Detection of HPV42 in 100% (*n* = 11/11) of DPA samples. ACC, adenoid cystic carcinoma; AFX, atypical fibroxanthoma; AS, angiosarcoma; BPDCN, blastic plasmacytoid dendritic cell neoplasm; DM, desmoplastic melanoma; DTE, desmoplastic trichoepithelioma; EPC, eccrine porocarcinoma; ES, eccrine spiradenoma; LM, lentigo maligna melanoma; MAC, microcystic adnexal carcinoma; PM, pediatric melanoma; PPT, proliferating pilar cystic tumor; SAC, spiradenocarcinoma; SC, sebaceous carcinoma; SGC, sweat gland carcinoma; SKCM, cutaneous melanoma. Viral prevalence: proportion of samples with detected virus in any given tumor type. Abundance score: mean proportion of detected virus in any given tumor type. **C,** HPV42 abundance score in a cohort of 17 DPAs after sequence capture to DNA and RNA of 6,453 viruses. White diamond: HPV42 detected but below an abundance of 1%. ND, not determined. **D,** Top, Circos plot depicting HPV42–human genome breakpoints. Each arch represents one detected breakpoint. Bottom, lollipop presentation of integration breakpoints in the HPV42 genome. Each lollipop represents one breakpoint (Supplementary Table S4). **E,** FISH signal of HPV42 genome (magenta) and human genome integration site adjacent locus (yellow) in DPA nuclei (blue). White arrowheads indicate fusion signals. Scale bar, 2.5 μm. Individual channels and quantifications in Supplementary Fig. S4. **F,** Top, RNA-ISH staining for expression of early region HPV42 mRNA in lung metastasis of a DPA tumor (T) and the surrounding normal (N) tissue. Scale bar, 1 mm. Bottom, inset. Scale bar, 20 μm.

By exploiting the substantial amount of off-target reads in targeted sequencing data (3) and utilizing a metagenomic classifier with a custom database (Viral DB) covering all viruses (>6,000) infecting vertebrate hosts (4), we screened 214 samples from 19 skin tumor types for viral pathogens (Fig. 1A). We did not find viral sequences in most analyzed skin tumors except for two tumor types: MCV in Merkel cell carcinoma, confirming the feasibility of our approach, and, notably, human papillomavirus 42 (HPV42) in all (*n* = 11/11) digital papillary adenocarcinomas (DPA; Fig. 1B; Supplementary Fig. S1A).

DPA is an aggressive, metastasizing skin cancer that occurs predominantly on the fingers and toes of young males (Supplementary Table S1). Although DPA is thought to arise from eccrine stem cells, the molecular drivers are thus far unknown (Supplementary Fig. S1A; ref. 5). To validate our discovery of HPV42 in DPA, we enriched viral nucleic acids from tumor samples using a hybridization-based sequence capture assay (ViroCap; ref. 4) and confirmed the presence of HPV42 RNA and DNA (Fig. 1C; Supplementary Fig. S1B and S1C; Supplementary Tables S2 and S3). In all but one DPA sample, which harbored a deletion of the late HPV region, the sequencing reads covered the complete HPV42 genome (Supplementary Fig. S1D). In an extended cohort, we confirmed the presence of HPV42 in 96% of DPA samples using qPCR of tumor DNA (*n* = 45/47; Supplementary Fig. S1C; Supplementary Table S1).

HPV42 is currently classified as a “low-risk” HPV type of the Alpha-1 species (genus *Alphapapillomavirus*), which has not been associated with oncogenic transformation (Supplementary Fig. S2A; ref. 6). Similar to other nononcogenic, “low-risk” HPV types (e.g., HPV6 or 11), HPV42 causes benign warts and is only rarely found in cancer (7, 8). In contrast, oncogenic, “high-risk” HPV types, such as HPV16 or 18, have an established role in tumorigenesis (Supplementary Fig. S2A; refs. 9, 10) and are frequently integrated into the host genome, facilitating the high expression of the viral oncogenes E6 and E7 (Supplementary Fig. S2B; ref. 11). Notably, we identified and confirmed HPV42 integrations in six DPAs (*n* = 6/16, 37%) by mapping HPV42–human genome breakpoints by targeted and Sanger sequencing (Fig. 1D; Supplementary Figs. S2C and S3A–S3D and Supplementary Table S4). To investigate the breakpoints in DPA tumors at the single-cell level, we designed a fluorescence *in situ* hybridization (FISH) experiment using probes targeting the HPV42 genome and the human genome adjacent to the integration breakpoint, inferred from sequencing data (Supplementary Table S5). The magenta viral probe was visible as a single spot in the tumor cells that overlapped with the yellow human probe (Fig. 1E; Supplementary Fig. S4A and S4B). These fusion signals suggest a clonal expansion of cells with the integrated HPV42 virus. The viral oncogenes E6 and E7 always remained intact during the integration event, and in 44% of cases (*n* = 7/16), we found a disruption of the viral genes E1 and E2, which is important for the derepression of E6 and E7 (Fig. 1D; Supplementary Tables S4, S6, and S7; ref. 12).

To confirm the expression of HPV42 E6 and E7 in DPA tumors, we utilized RNA *in situ* hybridization (RNA-ISH) and found HPV42 transcripts in 23 of 24 DPA tumors but not in the surrounding normal tissues (Fig. 1F; Supplementary Table S1). HPV42 transcripts were predominantly expressed from the early rather than late region of the HPV42 genome, consistent with transcriptional patterns observed for HPV16-induced cervical cancers and attributable to an incomplete differentiation state of the infected cell (ref. 13; Supplementary Fig. S2B). Altogether, these data suggest that HPV42 plays a central role in the pathogenesis of DPA.

### HPV42, Previously Classified as a Nononcogenic HPV, Induces Transformation *In Vitro* and Tumor Growth *In Vivo*

**Figure 2.**
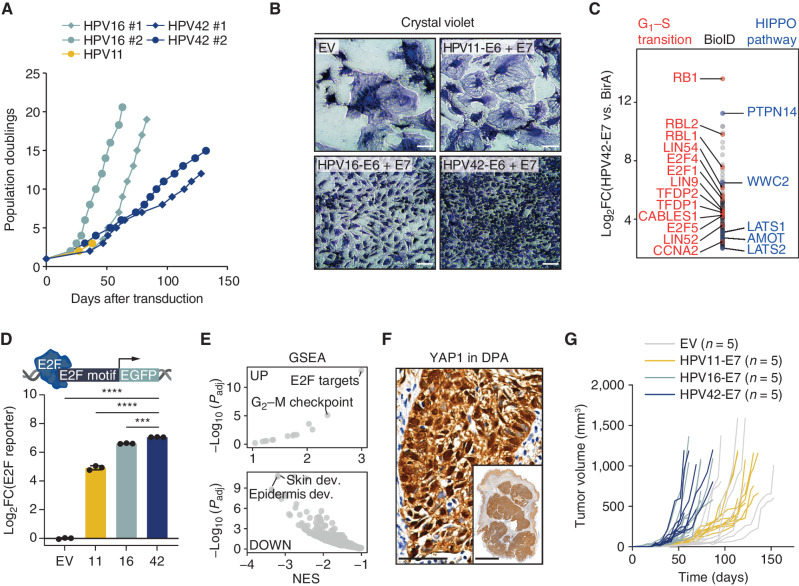
HPV42 induces transformation *in vitro* and tumor growth *in vivo*. **A,** Growth curves of HPKs transduced with the full genome of HPV11 (low-risk), HPV16 (high-risk), and HPV42. **B,** Crystal violet staining of MEF cells at day 14 after transduction. Scale bars, 100 μm. **C,** HPV42-E7 protein interaction partners identified in BioID proximity-labeling experiment. Pathway members of gene ontology terms G_1_–S transition (red) and HIPPO pathway (blue) are labeled. FC, fold change. **D,** E2F reporter assay in U2OS cells transduced with E7 proteins of HPV11, 16, or 42. ***, *P* < 0.001; ****, *P* < 0.0001 (one-way ANOVA, Tukey multiple comparisons test). **E,** Gene set enrichment analysis (GSEA) of the Molecular Signatures Database (MSigDB) hallmark gene set (top) and MSigDB curated gene set (bottom) comparing HPV42-E7 versus EV-transduced HPK cells. dev., development; NES, normalized enrichment score. **F,** IHC for YAP1 in a DPA tumor. Scale bar, main: 50 μm; inset: 3 mm. **G,** Individual tumor growth of EV- or E7 of HPV11-, HPV16-, or HPV42-transduced HaCaT cells in NSG mice. Each group consists of 5 mice (i.e., 10 tumors).

A key feature of oncogenic HPVs is the ability to extend the proliferative life span of primary cells such as human primary keratinocytes (HPK) or mouse embryonic fibroblasts (MEF; refs. 14–16). To functionally assess the oncogenic activity of HPV42, we transduced HPKs with the full genome of non-oncogenic HPV11, oncogenic HPV16, and HPV42. Although HPK-HPV11 cells stopped proliferating, HPK-HPV16 and HPK-HPV42 cells showed an extended proliferative life span (Fig. 2A; Supplementary Fig. S5A–S5C). Similarly, coexpression of only the E6/E7 oncoproteins of HPV42 and HPV16, but not of HPV11, promoted proliferation and prolonged the life span of HPKs and MEFs (Fig. 2B; Supplementary Fig. S5D–S5F).

To facilitate transformation, oncogenic HPVs utilize their E6 and E7 oncoproteins to alter cell-cycle progression (17, 18) and differentiation programs (19). The main target of the E6 protein of oncogenic HPVs is p53, which is polyubiquitinated and proteasomally degraded upon recruitment of the ubiquitin ligase E6AP (17, 20). Interestingly, we found that HPV42-E6 indeed strongly binds E6AP (Supplementary Fig. S5G and S5H; Supplementary Table S8), but in contrast to HPV16, p53 was not degraded and p53 targets remained, also after DNA damage induction, unchanged (Supplementary Fig. S5I–S5L). We next focused on the E7 protein, which, when derived from oncogenic HPVs, is sufficient to transform cells (21, 22). We expressed E7 of HPV11, 16, and 42 in HPK and MEF cells, and whereas empty vector (EV) or HPV11-E7–transduced cells entered senescence, cells with HPV42-E7 and HPV16-E7 showed an extended proliferative life span (Supplementary Fig. S6A–S6D).

To assess the interaction partners of HPV42-E7, we performed a proximity-labeling experiment identifying pRB as the top interaction partner together with several other proteins important for G_1_–S phase transition, such as RBL1, RBL2, and E2F family proteins (Fig. 2C; Supplementary Table S9). The RB family proteins (pRB, RBL1, and RBL2) are bound by oncogenic E7 via a conserved LxCxE motif (18, 21, 23). Notably, we found that the aspartic acid (D) upstream of the LxCxE motif, which has been shown to enhance pRB binding in oncogenic HPVs, is conserved in HPV42 (ref. 23; Supplementary Fig. S6E). Compared with HPV11, E7 of HPV42 and HPV16 indeed bound more strongly to pRB (Supplementary Fig. S6F), leading to equally robust activation of E2F signaling, an important indicator of functional disruption of pRB (Fig. 2D). Transcriptomic profiling identified E2F targets as the top upregulated gene class in HPK cells transduced with E7, which is in line with the proposed release of E2F transcription factors from RB family proteins by E7 interference (ref. 24; Fig. 2E; Supplementary Fig. S6G).

The second strongest binding partner of HPV42-E7 was the tumor suppressor PTPN14, together with several PTPN14-regulated components of the HIPPO pathway (Fig. 2C; Supplementary Table S9). We found that HPV42-E7, most likely involving the UBR4/p600 ubiquitin ligase (19), leads to the degradation of PTPN14, which promotes the persistence of dedifferentiated, HPV-infected cells in the basal layer of the skin (25) and is necessary for the transforming ability of oncogenic E7 proteins (ref. 26; Supplementary Fig. S6H). We found that PTPN14 loss leads to a downregulation of noncanonical HIPPO targets (Supplementary Fig. S6I), including skin/epidermis development–related pathways (Fig. 2E; Supplementary Fig. S6J), and downregulation of skin differentiation markers identified by quantitative mass spectrometry (MS) in HPV42-transduced cells (Supplementary Fig. S6K). We also observed predominantly nuclear localization of YAP1 in DPA tumors, indicating reduced noncanonical HIPPO signaling (Fig. 2F). Altogether, our data suggest that the inactivation of noncanonical HIPPO signaling by targeting PTPN14 for degradation plays a role in HPV42-driven DPAs.

To assess the oncogenic potential of HPV42-E7 *in vivo*, we expressed the E7 protein in HaCaT cells, a spontaneously immortalized human keratinocyte line (27, 28). We found that expression of E7 of HPV16 or HPV42 was sufficient to robustly induce tumor growth *in vivo* within 50 days, underlining the oncogenic potential of HPV42-E7 (Fig. 2G; Supplementary Fig. S6L). Collectively, our results suggest that HPV42 induces transformation *in vitro* and tumor growth *in vivo*.

### HPV42 Is Specifically Found in DPA

Considering the high prevalence of HPV42 in DPA and its oncogenic properties, we systematically screened for HPV42 and other HPVs in common cancers. We found that only ∼32% (*n* = 62/193) of HPVs and neither HPV42 nor other oncogenic HPVs (e.g., HPV45, 33, 52) are included in the NCBI Reference Sequence Database (RefSeq; Supplementary Fig. S7A–S7C), which is commonly used for metagenomic classification (1, 29). Thus, we generated an index based on the curated Papillomavirus Episteme database (PaVE; ref. 30) and assessed raw RNA sequencing (RNA-seq) data of 10,078 tumor samples across 32 cancer types from The Cancer Genome Atlas (TCGA) project. We detected HPVs in 3% of TCGA samples (*n* = 361/10,078; Supplementary Fig. S7D and Supplementary Table S10), including 93% of cervical cancer cases [cervical squamous cell carcinoma (CESC); *n* = 282/301] and 12% of head and neck cancer [head and neck squamous cell carcinoma (HNSCC); *n* = 62/498]. We also found HPV in rare cases of colon, urothelial, soft tissue, bladder, kidney, and lung cancers (Supplementary Fig. S7D and S7E). Notably, we did not identify HPV42 in any of the 10,078 TCGA tumors, indicating a cell lineage–specific role of HPV42-driven transformation in DPA (Supplementary Fig. S7D and S7E).

### All Oncogenic HPVs Induce a Germ Cell–like Transcriptional Program Conserved throughout HPV-Driven Cancers

**Figure 3.**
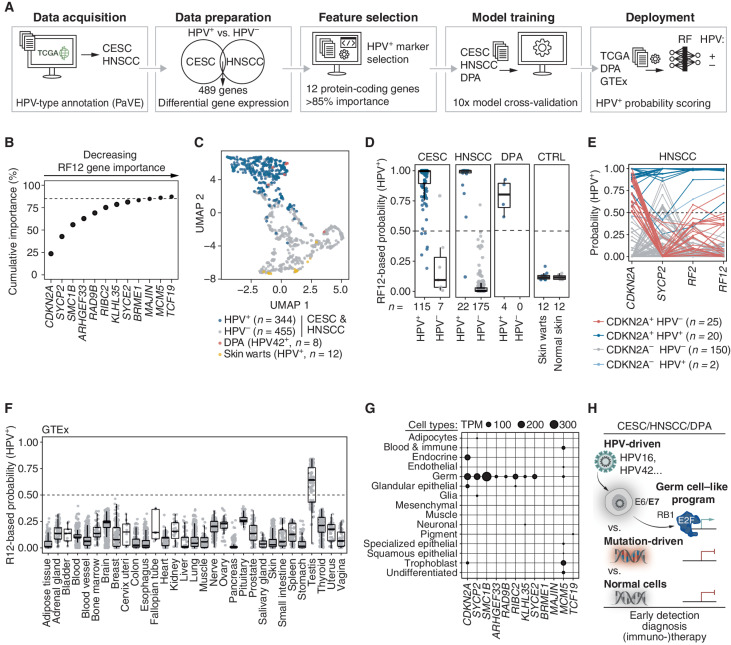
Oncogenic HPVs induce a germ cell–like transcriptional program conserved throughout HPV-driven cancers. **A,** Overview of the random forest (RF) feature selection and machine learning procedure to identify a transcriptional fingerprint of HPV-driven oncogenesis. CESC and HNSCC expression data were annotated with HPV status (data acquisition), commonly differentially expressed genes between HPV^+^ and HPV^−^ were identified (data preparation), and 12 protein-coding signature genes (RF12) were identified (feature selection). Four different machine learning models were trained with RF12 on a subset of DPA, CESC, and HNSCC samples (model training); the model with the best hyperparameters was evaluated using the withheld sample subset and used to classify Genotype-Tissue Expression (GTEx), skin warts, or normal skin expression data (deployment). **B,** Contribution of RF12 signature genes to cumulative feature importance (%) to discriminate between HPV^+^ and HPV^−^ tumor samples. **C,** Uniform manifold approximation and projection (UMAP) dimensionality reduction of RF12 expression in DPA, CESC, HNSCC, and skin warts. **D,** HPV^+^ probability scores were calculated by RF12 for CESC, HNSCC, DPA, skin warts, and normal skin. For CESC, HNSCC, and DPA, the test set samples are displayed. **E,** HPV^+^ probability scores for HNSCC calculated for *CDKN2A* and *SYCP2* alone, the combination of *CDKN2A* and *SYCP2* (RF2), and RF12. **F,** HPV^+^ probability scores were calculated by RF12 for 31 normal tissues obtained from the GTEx database. **G,** Expression levels of RF12 genes in 15 cell types obtained from Human Protein Atlas. **H,** Schematic model of the germ cell–like program in HPV-driven cancer.

We next used a machine learning approach to investigate whether HPV-driven cancers share a common transcriptional program that reveals more about the biology of HPV-driven cancers and could be exploited diagnostically and therapeutically. We annotated expression data of CESC and HNSCC from TCGA with the HPV status and identified 489 differentially expressed protein-coding genes between HPV^+^ and HPV^−^ cases across both cancer types (Fig. 3A; Supplementary Table S11). Feature selection using a random forest (RF) algorithm on the differentially expressed genes revealed a signature of 12 protein-coding genes (RF12; Fig. 3B; Supplementary Fig. S8A). The RF12 signature was sufficient to separately cluster HPV^+^ and HPV^−^ tumors across CESC and HNSCC regardless of their tissue origin and the oncogenic HPV type (Fig. 3C). Importantly, RF12 was also conserved in DPA, which clustered with the HPV^+^ tumors, but was absent in HPV-associated skin warts, which clustered with HPV^−^ tumors (Fig. 3C and D; Supplementary Fig. S8B).

To assess the specificity and sensitivity of RF12 in identifying HPV-driven tumors, we trained four machine learning classifiers on a training set of randomly selected tumor samples (*n* = 484, 60%) from CESC, HNSCC, and DPA (Fig. 3A). By applying the models to a test set withheld from training (*n* = 323, 40%), we found that all models achieved excellent performance (AUC >0.99) in predicting HPV as the cancer driver (Supplementary Fig. S8C). RF12 identified HPV-driven tumors with an accuracy of 94% in CESC, 99% in HNSCC, and 100% in DPA (Supplementary Fig. S8D) and scored equally in independent cohorts of HNSCC and CESC tumors (Supplementary Fig. S8E). We found that RF12 also scored in precancerous lesions of the cervix [high-grade squamous intraepithelial lesion (HSIL)], but not in HPV-associated skin warts and matched normal tissue (Fig. 3D; Supplementary Fig. S8F). Collectively, RF12 serves as an HPV fingerprint specific for HPV-driven oncogenic transformation, which is present in DPA tumors and supports that HPV42 is the oncogenic driver of DPA.

In the clinic, the accurate identification of HPV in HNSCC and CESC tumors and its precancerous lesions plays an important role (5). IHC for upregulation of p16/*CDKN2A*, the gene with the highest predictive contribution within RF12, is an established surrogate marker for oncogenic HPV infection (Supplementary Fig. S8A and S8B). However, p16/CDKN2A IHC lacks specificity, leading to diagnostic and therapeutic shortcomings. For example, ∼10% of patients with HPV^−^ oropharyngeal squamous cell carcinoma, a common form of HNSCC, are p16/CDKN2A^+^ but not HPV-driven. Therefore, these patients are incorrectly assigned to the more prognostically favorable group of HPV^+^ patients, putting these patients at risk of undertreatment (31, 32).

To improve the accurate identification of HPV^+^ tumors, we investigated *SYCP2*. *SYCP2*, a gene that in germ cells is important for meiotic prophase, has the second-highest predictive contribution within the RF12 signature and shows specific expression in HPV-driven tumors (Fig. 3B; Supplementary Fig. S8A). We found that the combination of *SYCP2* with *CDKN2A* improved HPV status assessment in HNSCC from 85% with *CDKN2A* alone to 97% accuracy (Fig. 3E; Supplementary Fig. S8G). Also, in DPAs, *CDKN2A* expression or p16/*CDKN2A* staining alone correctly identified HPV42-positive cases only in 70% to 75% of cases; however, the combination with *SYCP2* improved HPV status assessment to 100% accuracy (Supplementary Fig. S8G and S8H). Importantly, we confirmed in HPV^+^ DPA and CESC tumors that IHC is sufficient to assess *SYCP2* expression (Supplementary Fig. S8I and S8J). Altogether, these data suggest that adding a single gene, *SYCP2*, to routine diagnostic IHC of p16/*CDKN2A* could provide a reliable, cost-effective, and easily accessible method for accurately assessing HPV status in the clinic.

When investigating RF12 in normal tissues retrieved from the Genotype-Tissue Expression portal (GTEx; ref. 33), we found that RF12 is absent in 30 normal tissues but strongly scored in testis (Fig. 3F). Interestingly, we found that the majority of RF12 genes are highly and exclusively expressed in cells of the germ cell lineage and 9 of 12 genes play a role in processes specific for germ cells, such as meiosis (Fig. 3G; Supplementary Fig. S9A and S9B). Notably, this germ cell–enriched expression pattern is not limited to RF12 but expands to the 197 protein-coding genes we found commonly upregulated in HPV^+^ tumors (Supplementary Fig. S9B–S9D). We found that the E2F family transcription factor motif was the top enriched motif in the genes upregulated in HPV-driven tumors (Supplementary Fig. S9E–S9G). Altogether, our data suggest that the E7 oncoprotein of oncogenic HPVs interferes with the pRB–E2F axis in cancer cells and evokes a conserved germ cell–like program in HPV-driven tumors, which, even when reduced to the top two genes (*CDKN2A* and *SYCP2*), accurately distinguishes HPV-driven from mutation-driven tumors and outperforms established clinical markers (Fig. 3H).

## DISCUSSION

In summary, our study reveals HPV42 infections as a uniform driver mechanism of DPA, an aggressive skin cancer with thus-far unknown etiology that lacks mechanism-based therapeutic options. By functionally characterizing HPV42, previously considered a “low-risk” virus, our study adds a new member to the short list of tumorigenic viruses in humans. Prophylactic HPV vaccination is a remarkably effective preventive measure for HPV-associated diseases, but current vaccines immunize against only the most common (2–9) HPV types (34). Our data provide a rationale for including additional HPV types in the development of new vaccines and highlight the potential of pan-HPV vaccines (35, 36).

It is notable that the cell context in which HPV42 is present may determine the full oncogenic potential of HPV42. In the keratinocyte cell lineage of the mucosal (ano-)genital region, HPV42 can only lead to warts (thereby recapitulating features of “low-risk” HPV types), whereas in the eccrine lineage (sweat glands), which are most abundant on the hands and the feet, it causes oncogenic transformation leading to DPAs (recapitulating features of “high-risk” HPV types). In addition to this cell lineage–specific oncogenic potential of HPV42, it is also intriguing that HPV16 and HPV18, which dominate cervical and head cancers, are absent in DPAs.

The HPV-induced germ cell–like program we find in all HPV-driven cancers not only has diagnostic implications but also represents a vulnerability that could be exploited immunotherapeutically, which has proven difficult in HPV-driven cancers but holds promise for durable tumor control if successful. The top genes of the HPV-induced germ cell–like program have immunogenic properties, as they are only expressed in HPV^+^ cancers and immune-privileged germ cells but not in healthy somatic tissues and are predicted to be presented on common MHC class I molecules (Supplementary Fig. S9H). These properties make them promising targets for therapeutic cancer vaccines to induce an antitumor T-cell response, particularly in combination with vaccines involving HPV antigens E6 and E7 that are in clinical trials (NCT04405349 and NCT04287868), or as targets for engineered T cells in combination with T cells targeting E7 (37).

## METHODS

### Targeted Sequencing of Rare Skin Tumors

Sequencing of IMPACT tumors (*n* = 208) and pooled normal samples was performed as previously described (38). For whole-exome sequencing (WES) of six DPA tumor samples (Supplementary Table S1), genomic DNA was extracted from 10-μm-thick sections of formalin-fixed, paraffin-embedded (FFPE) tumor tissues. Sections were deparaffinized and manually macrodissected, and genomic DNA was isolated using the QIAamp DNA Mini Kit (#56304, Qiagen) according to the manufacturer's instructions. WES libraries were generated with the SureSelectXT Library Preparation Kit following the SureSelectXT Target Enrichment System for Illumina B.2 protocol (April 2015). Samples were sequenced on an Illumina HiSeq4000 in 150-bp, paired-end mode. The study complies with the declaration of Helsinki and was performed in accordance with the guidelines set forth by the ethics committee of the University of Duisburg-Essen under the Institutional Review Board number 15-6661-BO; written informed consent was obtained from each patient.

### Rare Skin Cancer Virus Detection Pipeline

Unprocessed reads of targeted sequencing data from 214 skin tumor samples (WES, *n* = 6; IMPACT, *n* = 208; Supplementary Table S1) were analyzed with the Centrifuge metagenomics classifier (RRID:SCR_016665, v1.0.4) using a custom-build reference index comprising 6,453 viruses (Viral DB; ref. 4). Results tables were filtered for viruses with a minimum abundance of 1% and at least one unique genome aligning read. A collection of matched normal samples derived from the IMPACT cohort was analyzed in the same way, and viruses present in the normal sample cohort were excluded from further analysis in the tumor cohort. For each virus exclusive to the tumor cohort, a mean abundance score, as well as the viral prevalence, was calculated.

### Virus Sequence Capture Assay (ViroCap)

Total RNA was extracted from FFPE sections using the Qiagen AllPrep DNA/RNA FFPE kit (#80234, Qiagen), and rRNA was depleted using the NEBNext rRNA Depletion Kit (#E6310, NEB). Sequencing libraries were prepared with the NEBNext Ultra II RNA Library Kit (#E7760, NEB) and enriched for viral sequences using the Roche SeqCap EZ Choice Probes using the ViroCap targeted sequence capture panel (4).

Total DNA was extracted from FFPE sections using the Qiagen AllPrep DNA/RNA FFPE kit (#80234, Qiagen). Sequencing libraries were prepared with the KAPA Hyper Prep Kit (#KK8504) and enriched for viral sequences using the Roche SeqCap EZ Choice Probes using the ViroCap targeted sequence capture panel (4). Samples were sequenced on an Illumina HiSeq4000 in 75-bp, paired-end mode. Unprocessed reads of virus sequence capture assay were analyzed using the Centrifuge metagenomics classifier (RRID:SCR_016665, v1.0.4) as described above. For each sample (DNA/RNA), abundance scores representing each detected viral species were calculated (Supplementary Tables S2 and S3).

### Alpha Genus Phylogenetic Tree

L1, L2, E1, and E2 protein sequences of 65 human *Alphapapillomavirus* strains were downloaded from PaVE (RRID:SCR_016599), and each protein family was aligned with mafft (-linsi method; RRID:SCR_011811). For phylogenetic analysis, the aligned sequences from each strain were fused, and a maximum likelihood tree was inferred with iqtree2 (RRID:SCR_021163, v.2.1.3), with standard model selection using ModelFinder (39) and ultrafast bootstrap (UFBoot2) support values (40). The tree was visualized in iTOL (RRID:SCR_018174, v6). According to PaVE (RRID:SCR_016599), HPV177 is classified as Alpha-7 but is placed in the clade of the Alpha-11 group in the phylogenetic tree.

### Viral Genome Analysis

Unprocessed reads from the virus sequence capture assay were processed with the Nextflow nf-core/viralrecon pipeline (https://nf-co.re/viralrecon, v2.4.1). Briefly, adapters were trimmed with fastp (RRID:SCR_016962, v0.23.2), reads aligned with Bowtie2 (RRID:SCR_016368, v2.4.4), and genomes sorted and indexed with SAMtools (RRID:SCR_002105, v1.14). Duplicates were marked with picard (RRID:SCR_006525, v2.26.10). Variant calling and consensus sequence generation were performed with BCFTools (RRID:SCR_002105, v1.14) and BEDTools (RRID:SCR_006646, v2.30.0; Supplementary Tables S6, S7, S12, and S13). Consensus genomes and reference genomes were compared using BLAST+ (ref. 41; v2.8.1; Supplementary Table S13). Viral genome coverage statistics were calculated using mosdepth (RRID:SCR_018929, v0.3.3; Supplementary Table S3). Genome coverage was visualized using the ggplot2 package (RRID:SCR_014601, v3.3.5) in R (https://www.r-project.org/, v.4.1.1).

### HPV42–Human Genome Breakpoint Identification and Validation

For integration site calling, reads were aligned with bwa (RRID:SCR_010910, v.0.7.17) to a fusion genome of the human genome (hg38) and the HPV42 PaVE reference sequence. Duplicates were marked, alignments were sorted, and BAM files were indexed with sambamba (ref. 42; v0.6.6). Integration sites were called using the Manta structural variant caller (ref. 43; v1.6) in high-sensitivity calling mode (minEdgeObservations = 2, minCandidateSpanningCount = 2). Structural variants detected within the HPV42 genome are reported in Supplementary Table S14, and all HPV42–human genome breakpoints with a read support of at least two spanning- and two split-reads supporting the alternative over the reference allele were further analyzed and are reported in Supplementary Table S4. Based on assembled contig sequences covering the HPV42–human genome breakpoints, primers were designed for validation by PCR followed by Sanger sequencing (Supplementary Table S4). HPV42-human genome breakpoints within 500 kb were inferred as one integration event (44–46). HPV42–human genome breakpoints were visualized using the circlize (RRID:SCR_002141, v0.4.13), trackViewer (ref. 47; v1.32.1), sangerseqR (ref. 48; v1.32), and Gviz (ref. 49; v1.40.1) packages in R (v.4.1.1).

### FISH

FISH was performed on 4-μm FFPE tumor sections using probes targeting HPV42 or a 10,000-bp region on the human genome adjacent to the integration site. Primary probes were designed using the OligoMiner package (50) using a fusion genome of HPV42 (PaVE) and human (hg38). Briefly, oligonucleotides were identified using the blockParse.py script (-l 36, -L 42 -t 42 -T 46 -S 2), candidates aligned using Bowtie2 (RRID:SCR_016368, v2.4.4; –no -hd -t -k 100 –very-sensitive-local) to the fusion genome, unique probes identified using outputClean.py (-T 42 -p 0.5), and high-abundance kmers filtered using kmerFilter.py (options -m 18 -k 5; Supplementary Table S5). Primary oligonucleotide probes containing handles for the annealing of secondary fluorescent oligonucleotides were purchased from IDT as oPools Oligo Pools with a concentration of 10 pmol/oligonucleotide. Secondary oligonucleotide probes 5′-ATTO647-labeled CACCGACGTCGCATAGAACGGAAGAGCGTGTG (HPV42), 5′-ATTO565-labeled CGAGCCAGGTCATCCTAGCCCATACGGCAATG (#170) and 5′-ATTO565-labeled TAGCGCAGGAGGTCCACGACGTGCAAGGGTGT (#184) were purchased from IDT.

Deparaffinized, protein-digested, and dehydrated tissue slides were preincubated for 20 minutes at 60°C in 2× SSCT (0.3 M NaCl, 0.03 M NaCitrate, 0.1% Tween-20) with 50% formamide (118143000, Sigma-Aldrich) and then incubated with a hybridization cocktail containing 2× SSCT, 50% formamide, 10% (w/v) dextran sulfate, 10 μg RNase A, 20 pmol/L primary oligonucleotide probe, and 40 pmol/L secondary oligonucleotide probe for 5 minutes at 95°C, followed by overnight incubation at 42°C in a humidified chamber as reported before (51). Slides were washed for 15 minutes at 60°C in 2× SCCT, followed by washes in 2× SSCT and 0.2× SSC for 10 minutes each at room temperature. Tissue sections were stained for 5 minutes in 0.2× SSC DAPI (1 μg/mL) solution in the dark, washed for an additional 3 minutes in 2× SSC, and mounted with Prolong Gold Antifade reagent (#P36970, Thermo Fisher Scientific).

FISH-stained tissue sections were acquired at room temperature on a Zeiss LSM980 microscope with the Airyscan2 detector, operated by the ZEN 3.3 (RRID:SCR_013672, blue edition) software. A 63 × 1.4NA oil-immersion DIC plan-apochromat objective (Zeiss) and 405/561/639-nm laser excitation (imaged sequentially with a track switch after every Z-Stack) were used to acquire Z-Stacks with 18 frames (33.7 μm × 33.7 μm) within a range of 3.2 μm (0.18 μm interval). Image sampling was conducted with a pixel size of 0.04 μm, a sampling speed of 0.83 μs per pixel, and the 8× averaging and calculation of the mean intensity per frame.

For quantitative analysis of fusion signals, 9 to 11 fields of view (FOV) per tissue slide and condition were recorded. Per FOV, a median of 5 (range, 3–9) nuclei with HPV42 and human signals in the same nucleus were analyzed using Fiji [(RRID:SCR_002285), ImageJ v2.3, RRID:SCR_003070]. Detection, colocalization, and quantification were performed using the ComDet (https://github.com/ekatrukha/ComDet, v.0.5.3) plug-in for ImageJ.

### RNA-ISH

*In situ* detection of HPV42 mRNA was performed by the Vienna BioCenter Core Facilities (VBCF) Histology Facility using RNAscope 2.5 HD Assay-Red (#322360, Advanced Cell Diagnostics) using probe #548288 (RNAscope 2.5 LS Probe-V-HPV42-O1) according to the manufacturer's protocol. The 5-μm-thin FFPE sections were pretreated for 15 minutes with Target Retrieval Solution and for 30 minutes with Protease Plus. After hybridization, the mRNA signal was amplified and visualized with an alkaline phosphatase–based red substrate giving red dots as a positive signal. Housekeeping gene *PPIB* was used as a positive control to ensure mRNA quality, whereas the bacterial Diaminopimelate (dapB) gene was used as a negative control.

### RT-qPCR Detection of HPV42

HPV42 was detected in cellular DNA by qPCR with amplicons in HPV42-E6 (F: ACAGCCACGCACATTATACCA; R: AAATGGTACGCGAGCACCTC) and HPV42-E7 (F: AACGTGTGAGACACCCATTGA; R: ATGTCCTGTTTGGCTTGGTCA). The genomic LINE1 locus (F: CGCTTTTCAGACCGGCTTAAG; R: AGATTCCGTGGGCGTAGGA) was used for data normalization. qPCRs were performed with the Luna Universal qPCR Master Mix (#M3003, New England BioLabs) on a Bio-Rad CFX384 Real-Time Cycler.

### Expression Profiling of FFPE DPA Tumor Samples

Total RNA was extracted from FFPE DPA tumor samples using the MagMAX FFPE DNA/RNA Ultra Kit (#A31881, Life Technologies Limited) according to the manufacturer's instructions and eluted in nuclease-free water. Concentration was determined with the spectrophotometer/fluorometer DeNovix DS-11 Fx and assessed for RNA integrity on a Fragment Analyzer System (Agilent). RNA-seq (3′-end) mRNA sequencing libraries (QuantSeq) were prepared from 500 ng RNA using the QuantSeq 3′ mRNA-Seq Library Prep Kit FWD for Illumina (Lexogen) and PCR Add-on Kit for Illumina kits (020.96, Lexogen) according to the manufacturer's instructions. Sequencing was performed on an Illumina NovaSeq 6000 in 100-bp, single-end read mode. Analysis of QuantSeq data was performed with an in-house pipeline: Briefly, adapter and polyA sequences were clipped (bbdk, RRID:SCR_016965, v38.06), abundant sequences were removed with bbmap (RRID:SCR_016965, v38.06), and cleaned reads were aligned against the genome (hg38) with STAR (RRID:SCR_004463, v2.6.0c). Raw reads were mapped to 3′ untranslated region (UTR) annotations of the same gene and collapsed to gene level by Entrez Gene ID with featureCounts (RRID:SCR_012919, v1.6.2). For visualization of HPV42 genome QuantSeq read coverage, reads were aligned with HISAT2 (RRID:SCR_015530, v2.1.0) to a fusion genome of hg38 and the HPV42 PaVE (RRID:SCR_016599) reference sequence; genome coverage tracks were calculated with BEDtools (RRID:SCR_006646, v2.27.1) and visualized with SparK (ref. 52; v2.6.2).

### Generation of Full-Genome HPV-Transduced Keratinocytes

HPKs were isolated from human foreskin after circumcision upon written informed consent from the patients, which was approved by the ethics committee of the medical faculty of the University Tuebingen (6199/2018BO2) and performed according to the principles of the Declaration of Helsinki. HPKs were maintained in keratinocyte serum-free medium supplemented with recombinant human epidermal growth factor, bovine pituitary extract, and gentamicin (#15710064, Thermo Fisher Scientific). HPKs were transfected with 4 μg recircularized HPV genomes and 2 μg of pSV2-neo plasmid using Fugene HD (#E2311, Promega; ref. 53). Cells were selected with G418 (150 μg/mL, #ant-gn-1-1g, InvivoGen) in Dulbecco's modified Eagle's medium and Ham's F12 (3:1) supplemented with 5% (v/v) fetal bovine serum; 24 μg/L adenine, 0.4 ng/L hydrocortisone (#H0888, Sigma-Aldrich), 10 ng/L cholera toxin (#100B, List Biological Labs, Inc), 5 μg/L transferrin (#T1147, Sigma-Aldrich), 20 pmol/L 3,3′-5-triodo-L-thyronine, 5 ng/L epidermal growth factor (#E4127, Sigma-Aldrich) and 5 μg/L insulin (#91077, Sigma-Aldrich) and mitomycin C-treated NIH 3T3 J2 NHP until mock-transfected keratinocytes were dead (8–10 days; refs. 53, 54). G418-resistant colonies were expanded as pooled cultures. Cells were maintained continuously, and splitting dates and dilutions were recorded to generate growth curves. For short-term growth measurements, 1.5 × 105 HPV16 or 42-positive keratinocytes were seeded in 6-well dishes in the presence of growth-arrested NIH3T3 J2. At the indicated time points, fibroblasts were first removed by incubation with 0.5 mmol/L EDTA in PBS. Keratinocytes were detached by trypsinization, stained with trypan blue, and counted using an automated cell counter (Countess, Invitrogen).

### Generation of HPV E6/E7-Transduced Keratinocytes

For lentivirus production, HEK-293T (Lenti-X) cells were cotransfected with the lentiviral backbone expressing the E6 and E7 proteins under the control of an SFFV promoter (pTwist-SFFV-E6-P2A-E7-T2A-mCherry-IRES-PURO-WPRE), VSVG (RRID:Addgene_12259) as enveloping plasmid, and the packaging plasmid PAX2 (RRID: Addgene_12260) in standard medium with polyethyleneimine as previously reported (55). Twenty-four hours after transfection, the culture medium was changed to 1% fetal bovine serum (FBS) medium, and viral supernatant was collected after 24 hours and filtered through a 0.4-μm mesh. For transduction of HPK cells, 5 × 10^6^ HPK cells were seeded in a 60-mm plate and 24 hours after seeding incubated with viral supernatant for 6 hours. Another 24 hours later, cells were expanded into 100-mm plates and selected with 2 μg/mL puromycin. On day 3 after transduction, 5 × 10^6^ HPK cells were seeded in a 6-well plate. Cells were counted each third day and reseeded with a 1:3 split ratio. The remaining cells were plated for crystal violet (CV) staining. HEK-293T cells were purchased from Takara (Lenti-X 293T, #632180).

### Generation of HPV E6/E7-Transduced MEFs

Eight-week-old C57BL/6 female mice were euthanized 13.5 to 14.5 days after the appearance of the copulation plug by cervical dislocation to remove the uterine horns. Embryos within the uterine horn were collected by extraction of the embryonic sacs, beheaded, red tissue removed, and the remaining tissue minced into pieces of 1- to 2-mm size and resuspended in PBS. For each 1 mL of tissue suspension, 1 mL 0.25% trypsin was added, and the tissue was digested at 37°C for 10 minutes. The tissue suspension was further homogenized using a 21G needle, and the digestion was quenched by the addition of 5 mL growth medium (DMEM + 10% FBS). Cells were spun at 400 g for 5 minutes, plated in a growth medium, expanded the following day, and frozen in freezing medium (10% DMSO + 90% FBS). MEF (passage 2) cells were used for counting and staining assays. Two-hundred-thousand MEF cells were seeded per well in a 6-well plate. Twenty-four hours after seeding, cells were incubated with lentivirus encoding for the E6 and E7 proteins under the control of an SFFV promoter (pTwist-SFFV-E6/E7-IRES-PURO-WPRE) in the presence of 8 μg/mL polybrene and spun at 800 × *g* for 1 hour at 35°C. Viral supernatant was removed 24 hours after spinfection and replaced with fresh medium. On day 3 after transduction, 100,000 MEFs were seeded at equal transduction efficiency in a 6-well plate. Cells were counted each third day and reseeded with a 1:3 split ratio. The remaining cells were plated for CV staining. Cells were tested for *Mycoplasma* regularly.

### CV Staining

The cell culture medium was removed, and cells were washed twice with PBS and fixed for 5 minutes with 1.5% PFA in PBS. PFA was removed, and cells were washed 3× with PBS and stained with CV solution (0.5% CV, 1% formaldehyde, 1% MeOH) for 5 minutes at room temperature. Finally, the CV solution was removed, and cells were washed 3× with PBS.

### Viral Copy-Number Determination and Exonuclease V-Resistance Assay

Total cellular DNA was isolated by proteinase K digestion, followed by phenol/chloroform/isoamyl alcohol extraction and ethanol precipitation. HPV copy numbers were quantified in cellular DNA by qPCR detecting amplicons in HPV16 (HPV16-E6 F: GAGAACTGCAATGTTTCAGGACC, HPV16-E6 R: TGTATAGTTGTTTGCAGCTCTGTGC), HPV42 (HPV42-E7 F: ATGACCAAGCCAAACAGGAC, HPV42-E7 R: CAATATCCAGTGTGCCCAAA), and the cellular *ACTB* gene (ACTB F: GGAAGGTCCGTGCGAGG, ACTB R: GAGACGGAGCAGGTCCCA) and copy-number standards using a LightCycler 480 and the LightCycler 480 SYBR green I master mix (Roche Applied Science). The exonuclease V resistance assay was adapted from (56) with minor modifications: total cellular DNA (100 ng) was incubated in the presence or absence of 5U of exonuclease V (#M0345S, NEB) in 1× NEBuffer 4 supplemented with 1 mmol/L ATP for 60 minutes at 37°C. Then, the enzyme was inactivated for 10 minutes at 95°C. Finally, 10 ng of input DNA was measured by qPCR using primers for HPV16, HPV42, and *ACTB*.

### *In Vitro* p53 Degradation Assay

TP53 and N-terminally V5-tagged E6 proteins of HPV11, 16, and 42 were cloned under the control of an SP6 promoter flanked by Xenopus globin 5′ and 3′ UTRs. Plasmids were linearized and transcribed for 12 hours at 37°C (#AM1340, mMessage mMACHINE SP6 Transcription Kit). Template DNA was removed by the addition of 1μL DNAse (#AM2238, TURBO DNAse) for 15 minutes at 37°C. Transcribed mRNA was purified using RNeasy MinElute Cleanup Kit (#74204, Qiagen) according to the manufacturer's instructions. The E6 and p53 proteins were *in vitro* translated for 90 minutes at 30°C using an in-house rabbit reticulocyte system (57). The p53 degradation reactions were performed in 10-μL volumes by incubating 2 μL of p53 translation product with 5 μL of E6 translation products at 28°C for 2 hours as previously described (58) and analyzed by immunoblotting.

### DNA Damage–p53 Response Assay

U2OS (RRID:CVCL_0042, generously provided by Egon Ogris, Max Perutz Labs, Vienna, Austria) and N/TERT-1 (RRID:CVCL_CW92, generously provided by James Rheinwald, Department of Dermatology, Brigham and Women's Hospital, Boston, MA; ref. 59) cells transduced with E6 genes of HPV11, HPV16, HPV42, or EV (pTwist-SFFV-E6-IRES-PURO-WPRE) were treated with 1 μg/mL mitomycin C or DMSO for 18 hours as previously described (60). Cells were harvested and analyzed by immunoblotting and qPCR using the following primers: TP53 (F: GAGGTTGGCTCTGACTGTACC, R: TCCGTCCCAGTAGATTACCAC), BAX (F: CCCGAGAGGTCTTTTTCCGAG, R: CCAGCCCATGATGGTTCTGAT), MDM2 (F: CAGTAGCAGTGAATCTACAGGGA, R: CTGATCCAACCAATCACCTGAAT), FAS (F: AGATTGTGTGATGAAGGACATGG, R: TGTTGCTGGTGAGTGTGCATT), and HPRT1 (F: TGACACTGGCAAAACAATGCA, R: GGTCCTTTTCACCAGCAAGCT). Cells were kept at a low passage and tested for *Mycoplasma* regularly.

### Immunoblotting

Cells were lysed with RIPA buffer (#9806, Cell Signaling Technology) supplemented with cOmplete Protease Inhibitor Cocktail (#5056489001, Sigma-Aldrich) and HALT phosphatase inhibitor (#78427, Thermo Fisher Scientific). Lysates were sonicated and cleared by centrifugation at 14,000 g for 10 minutes at 4°C. According to the manufacturer's instructions, protein concentrations were determined with the Pierce BCA Protein Assay kit (#23225, Thermo Fisher Scientific). Immunoblotting was conducted according to standard protocols. The primary antibodies used for immunoblotting were as follows: anti-Vinculin (Sigma-Aldrich; cat. #V9131, RRID:AB_477629, 1:1,000), anti-E6AP (Santa Cruz Biotechnology; cat. #sc-166689, RRID:AB_2211807, 1:500), anti-p53 (Santa Cruz Biotechnology; cat. #sc-126, RRID:AB_628082, 1:500), anti-RPL3 (GeneTex; cat. #GTX124464, RRID:AB_11169700, 1:1,000), anti-RB1 (Santa Cruz Biotechnology; cat. #sc-102, RRID:AB_628209, 1:500), anti-PEZ (Santa Cruz Biotechnology; cat. #sc-373766, RRID:AB_10917236, 1:500), anti-p21 (Cell Signaling Technology; cat. #2947), anti-H3 (Abcam; cat. #ab1791, RRID:AB_302613, 1:5,000), anti–α-tubulin (Cell Signaling Technology; cat. #2125, RRID:AB_2619646), 1:1,000), anti-V5 (Sigma-Aldrich; cat. #V8012, RRID:AB_261888, 1:5,000), and anti-CDKN2A/p16INK4 (Abcam; cat. #ab108349, RRID:AB_10858268, 1:1,000). The secondary antibodies used were as follows: anti-rabbit IgG HRP-linked (Cell Signaling Technology; cat. #7074, RRID:AB_2099233, 1:5,000), anti-mouse IgG HRP-linked (Cell Signaling Technology; cat. #7076, RRID:AB_330924, 1:5,000), and anti-mouse IgG light chain-specific HRP-linked (Cell Signaling Technology; cat. #58802, RRID:AB_2799549, 1:5,000).

### E6/E7 coimmunoprecipitation

Coimmunoprecipitation (co-IP) of E6 and E7 proteins were performed as previously reported (61). U2OS cells (RRID:CVCL_0042) were transfected with plasmids encoding 3xV5-tagged E6 proteins and 3xMYC-tagged E7 proteins under the control of a constitutive SFFV promoter (pTwist-SFFV-E6-P2A-E7-T2A-mCherry-IRES-PURO-WPRE). For E6 protein co-IPs, U2OS cells were cotransfected with pLX313-TP53-WT (RRID:Addgene_118014). Twenty-four hours after transfection, cells were treated for 24 hours with 1 μmol/L of the proteasome inhibitor MG-132 (#S2619, Selleck Chemicals). Cells were washed with ice-cold PBS and lysed with 1 × Cell Lysis Buffer (#9803, Cell Signaling Technology). Lysates were cleared by centrifugation at 14,000 × *g* for 10 minutes at 4°C, and the lysate concentration was determined with the Pierce BCA Protein Assay kit (#23227, Thermo Fisher Scientific) according to the manufacturer's instructions. Lysates were incubated with α-V5 agarose beads (#A7345, Sigma-Aldrich) or α-MYC agarose beads (#A7470, Sigma-Aldrich) for 2 hours at 4°C and washed 3× in 1 × Cell Lysis Buffer. Confirmation of successful co-IP was conducted by immunoblotting according to standard procedures.

### E2F Reporter

U2OS cells (RRID:CVCL_0042) were transduced with a lentiviral backbone expressing the E7 genes of HPV11, 16, or 42 or EV under the control of an SFFV promoter (pTwist-SFFV-E7-IRES-PURO-WPRE) and selected for puromycin resistance. Transduced cells were transfected with plasmids expressing destabilized GFP (GFPd2) under the control of an E2F DNA-binding site containing promoter (62). GFPd2 expression was assessed by flow cytometry 48 hours after transfection. single-cell events (2 × 10^4^) were acquired per sample on a FACS LSRFortessa cytometer, and GFP-positive fractions were determined with the FlowJo10 software (RRID:SCR_008520).

### MS: Quantitative MS

cells (1 × 10^6^ cells) were washed twice with ice-cold PBS, and proteins were prepared for MS analysis using the iST-NHS kit (P.O.00030, PreOmics) together with the TMT10plex Isobaric Label Reagent Set (#90110, Thermo Fisher Scientific) according to the manufacturer's instructions.

### nanoLC-MS/MS Analysis

Samples were analyzed on an UltiMate 3000 RSLC nano system (Thermo Fisher Scientific) coupled to an Exploris 480 mass spectrometer equipped with a FAIMS pro interface and a Nanospray Flex ion source (Thermo Fisher Scientific). Peptides were loaded onto a trap column (PepMap Acclaim C18, 5 mm × 300 μm ID, 5 μm particles, 100 Å pore size, Thermo Fisher Scientific) at a flow rate of 25 μL/minute using 0.1% TFA as mobile phase. After 10 minutes, the trap column was switched in line with the analytical column (PepMap Acclaim C18, 500 mm × 75 μm ID, 2 μm, 100 Å, Thermo Fisher Scientific). Peptides were eluted using a flow rate of 230 nL/minute, starting with the mobile phases 98% A (0.1% formic acid in water) and 2% B (80% acetonitrile, 0.1% formic acid) and linearly increasing to 35% B over the next 120 or 180 minutes. This was followed by a steep gradient to 90% B in 5 minutes, which stayed there for 5 minutes and ramped down in 2 minutes to the starting conditions of 98% A and 2% B for equilibration at 30°C.

For tandem mass tag (TMT) experiments, the Orbitrap Exploris 480 mass spectrometer (Thermo Fisher Scientific) was operated in data-dependent mode, performing a full scan (m/z range, 350–1,200, resolution 60,000, normalized AGC target 1 × 10^6^) at three different compensation voltages (CV; 40, 55, 70), followed each by MS/MS scans of the most abundant ions for a cycle time of 1 second per CV. MS/MS spectra were acquired using a collision energy of 34, isolation width of 0.7 m/z, resolution of 45.000, fill time of 120 ms, normalized AGC target of 2 × 10^5^, and intensity threshold of 1 × 104. Precursor ions selected for fragmentation (including charge states 2–6) were excluded for 45 seconds.

### Data Processing Protocol for TMT Experiments

For peptide identification, the RAW files were loaded into Proteome Discoverer (RRID:SCR_014477, v2.5.0.400). All hereby created MS/MS spectra were searched using MSAmanda (ref. 63; v2.5.0.16129) against the human Uniprot reference database (2021-06-30, 20,531 sequences; 11,395,157 residues), supplemented with common contaminants. The following search parameters were used: Carbamidomethyl on cysteine was set as a fixed modification, oxidation on methionine, deamidation of asparagine and glutamine, carbamylation and 10-plex/16-plex TMT on lysine and on peptide-N-term were set as variable modifications. The peptide mass tolerance was set to ±10 ppm and the fragment mass tolerance to ±10 ppm. The maximal number of missed cleavages was set to 2, and the minimum peptide length was set to 7. The result was filtered to 1% false discovery rate (FDR) on PSM and protein level using the Percolator algorithm integrated in Thermo Proteome Discoverer. Additionally, a minimum MS Amanda score of 150 was applied on the PSM level, and proteins had to be identified by a minimum of two PSMs. The localization of the modification sites within the peptides was performed with the tool ptmRS, which is based on phosphoRS (64). Peptides were quantified based on Reporter Ion intensities extracted by the “Reporter Ions Quantifier”-node implemented in Proteome Discoverer. Proteins were quantified by summing unique and razor peptides. Protein-abundances-normalization was done using sum normalization. The statistical significance of differentially expressed proteins was determined using limma (RRID:SCR_010943).

### BioID Sample Preparation

Briefly, Flp-In T-REx HEK293 cells were grown in DMEM (Gibco) supplemented with 10% FBS (Sigma-Aldrich), GlutaMAX, and penicillin–streptomycin (P/S, 1×). Using the Flp-In system (Invitrogen), Flp-In T-REx HEK293 stably expressing BirA* alone (for control samples) or N- and C-terminally tagged HPV16 and 42 E6 and E7 viral proteins were generated cotransfecting pOG44 with each pcDNA5 FRT/TO BirA*Flag-viral bait protein sequence plasmid. After selection (DMEM + 10% FBS + P/S + 200 μg/mL hygromycin B), three independent replicates of two 150 cm^2^ plates of subconfluent (60%) cells were incubated for 24 hours in complete media supplemented with 1 μg/mL tetracycline (Sigma) and 50 μmol/L biotin (Thermo Fisher Scientific). Cells were collected and pelleted (2,000 rpm, 3 minutes) and washed twice with PBS, and dried pellets were snap-frozen. Each cell pellet was resuspended in 5 mL of lysis buffer [50 mmol/L Tris-HCl pH 7.5, 150 mmol/L NaCl, 1 mmol/L EDTA, 1 mmol/L EGTA, 1% Triton X-100, 0.1% SDS, 1:500 protease inhibitor cocktail (Sigma-Aldrich), 1:1,000 Turbonuclease (Accelagen)], incubated on an end-over-end rotator at 4°C for 1 hour, briefly sonicated to disrupt any visible aggregates, and then centrifuged at 45,000 × *g* for 30 minutes at 4°C. The supernatant was transferred to a fresh 15-mL conical tube. Twenty-five microliters of packed, preequilibrated Streptavidin Ultralink Resin (Pierce) were added, and the mixture was incubated for 3 hours at 4°C with end-over-end rotation. Beads were pelleted by centrifugation at 2,000 rpm for 2 minutes and transferred with 1 mL of lysis buffer to a fresh Eppendorf tube. Beads were washed once with 1 mL lysis buffer and twice with 1 mL of 50 mmol/L ammonium bicarbonate (pH = 8.3), and then transferred in ammonium bicarbonate to a fresh centrifuge tube and washed two more times with 1 mL ammonium bicarbonate buffer. Tryptic digestion was performed by incubating the beads with 1 μg MS-grade TPCK trypsin (Promega) dissolved in 200 μL of 50 mmol/L ammonium bicarbonate (pH 8.3) overnight at 37°C. The following morning, 0.5 μg MS-grade TPCK trypsin was added to the beads and incubated 2 additional hours at 37°C. Following centrifugation at 2,000 × *g* for 2 minutes, the supernatant was collected and transferred to a fresh Eppendorf tube. Two additional washes were performed with 150 μL of 50 mmol/L ammonium bicarbonate and pooled with the first eluate. The sample was lyophilized and resuspended in buffer A (2% ACN 0.1% formic acid). One third of each sample was analyzed per mass spectrometer run.

### BioID Data Acquisition

Samples were separated by online reversed-phase chromatography using a Thermo Scientific Easy-nLC1000 system equipped with a Proxeon trap column (75 μm ID × 2 cm, 3 μm, Thermo Scientific) and a C18 packed-tip column (Acclaim PepMap, 75 μm ID × 50 cm, 2 μm, Thermo Scientific). The digested peptides were separated using an increasing amount of acetonitrile in 0.1% formic acid from 2% to 30% for 2 hours at a flow rate of 300 nL/minute. A voltage of 2.4 kV was applied by the liquid junction to electrospray the eluent using the nanospray source. A high-resolution mass spectrometer (Q-Exactive, Thermo Scientific) was coupled to the chromatography system to acquire the 10 most intense ions of MS1 analysis (Top 10) in data-dependent mode. The MS analyses were performed in positive mode at a resolving power of 70,000 FWHM, using an automatic gain control target of 3 × 10^6^, and the default charge state was set at 2 and a maximum injection time at 120 ms. For full scan MS, the scan range was set between m/z 300 and 1,600. For ddMS², the scan range was between m/z 200 to 2000, 1 microscan was acquired at 17,500 FWHM, an AGC was set at 5 × 10^4^ ions and an isolation window of m/z 4.0 was used.

### BioID Data Analysis

The proteins were identified by comparing all MS/MS data with the Homo sapiens proteome database (Uniprot, release April 2021, Reviewed, comprising 20,360 entries + viral bait protein sequences added manually), using the MaxQuant software (RRID:SCR_014485, v1.5.8.3). The digestion parameters were defined using trypsin with two maximum missed cleavages. The oxidation of methionine and N-terminal protein acetylation were defined as variable modifications. The label-free quantification (LFQ) was done keeping the default parameters of the software. As for initial mass tolerance, 6 ppm was selected for MS mode, and 20 ppm was set for fragmentation data to match MS/MS tolerance. The identification parameters of the proteins and peptides were performed with an FDR at 1%, and a minimum of two unique peptides per protein. The LFQ values from the 20 control runs (BirA* alone samples from stable cell lines) were collapsed to the three highest values for each given ID. These three values were defined as the control group for comparison with viral bait proteins triplicates. The statistical analysis was done by Perseus software (RRID:SCR_015753, v1.6.2.3). Briefly, the LFQ intensity of each sample was downloaded in Perseus, and the data matrix was filtered by removing the potential contaminants, reversed, and only identified by site. The data were then transformed using the log_2_(*x*) function. Before statistical analysis, eight (four viral bait proteins, N-ter, C-ter BirA* tag for each) were defined with three replicates per group. Only preys with detected values in all three replicates of a given viral bait protein were kept for further analysis. Missing values were then replaced from the normal distribution separately for each column. Two-sample Student *t* test was then performed comparing all three biological replicates of each bait and condition against the three controls runs. High-confidence proximal interactors were defined by permutation-based FDR with a cutoff of 0.01. Supplementary Tables S8 and S9 show the average log_2_-fold change against control and the corresponding q-values for each bait and condition.

### RNA Extraction from HPK, N/TERT-1, and U2OS Cells

Total RNA isolation from cells was performed with an in-house paramagnetic bead-based purification protocol. Briefly, cells were lysed, incubated with functionalized paramagnetic beads, and washed to remove proteins. Beads were then treated with DNase I, and purified total RNA was eluted in nuclease-free water. RNA concentration was determined with the spectrophotometer/fluorometer DeNovix DS-11 Fx and assessed for integrity on a Fragment Analyzer System (Agilent).

### Hematoxylin and Eosin and IHC Staining

Sections were stained with hematoxylin (#6765002, Thermo Scientific) and eosin (#6766008, Thermo Scientific; H&E) for histologic examination using the Thermo Scientific Gemini AS Automated Slide Stainer. For IHC, FFPE tissues were sectioned at 2 μm (HM355S Rotary Microtome), deparaffinized, and rehydrated. Antigens were retrieved using EDTA retrieval (Tris-EDTA, pH 8.5, 0.05% Tween-20) at 100°C for 30 minutes. Endogenous peroxidase was quenched for 10 minutes in 3% H_2_O_2_ diluted in water and slides blocked with 10% BSA and 10% goat serum in TBST for 1 hour at room temperature. IHC with anti-YAP1 and anti-SYCP2 was performed using DAB chromogen and hematoxylin counterstain. The following antibodies against human epitopes were used: anti-YAP1 (Cell Signaling Technology; cat. #14074, RRID:AB_2650491, 1:200 in 2% TBST, 1 hour) and anti-SYCP2 (Atlas Antibodies; cat. #HPA062401, RRID:AB_2684752, 1:200 in 2% TBST, 2 hours).

### QuantSeq Analysis of HPV/E7-Transduced HPKs

HPKs were washed in PBS, and cell pellets were snap-frozen. RNA extraction was performed using an in-house total RNA magnetic beads–based purification protocol. RNA-seq (3′-end) mRNA sequencing libraries (Quantseq) were prepared from 500 ng RNA using the QuantSeq 3′ mRNA-Seq Library Prep Kit FWD for Illumina (#015.96, Lexogen) and PCR Add-on Kit for Illumina kits (#020.96, Lexogen) according to the manufacturer's instructions. Sequencing was performed on an Illumina Novaseq 6000 in 100-bp, single-end mode. Analysis of QuantSeq data was performed with an in-house pipeline. Briefly, adapter and polyA sequences were clipped, and abundant sequences were removed with bbmap (RRID:SCR_016965, v38.06). Cleaned reads were aligned against the genome (hg38) with STAR (RRID:SCR_004463, v2.6.0c). Raw reads were mapped to 3′ UTR annotations of the same gene and collapsed to gene level by Entrez Gene ID with featureCounts (RRID:SCR_012919, v1.6.2). Differentially expressed genes were calculated using DESeq2 (RRID:SCR_015687, v1.18.1). Pathway enrichment analysis was performed using g:Profiler (RRID:SCR_006809) and Metascape (RRID:SCR_016620). Gene set enrichment analysis (65, 66) was performed using the FGSEA (RRID:SCR_020938, v1.19.2). Gene-based motif analysis was performed using Homer (RRID:SCR_010881, v4.11) with the findMotifs.pl script and default settings.

### *In Vivo* Tumor Growth of E7-Transduced HaCaT Cells

HaCaT cells (RRID:CVCL_0038) were transduced by spinfection (30 minutes, 800 × *g*, 37°C) with lentiviral backbones expressing the E7 gene of HPV11, HPV16, or HPV42 or EV under the control of an SFFV promoter. Transduced cells were selected with puromycin. HaCaT cells (2 × 10^6^) mixed with an equal volume of matrigel (#3562377, Corning) were injected subcutaneously into the left and right flanks of 5- to 8-week-old sex-randomized NSG mice. NSG (NOD-scid IL2R gamma null) mice were purchased from The Jackson Laboratory (strain 005557). Prior to tumor injection, the mice were injected intraperitoneally with analgesic ketamine (100 mg/kg), xylazine (10 mg/kg), and acepromazine (3 mg/kg). Tumor size was monitored by tumor measurement with an electronic caliper. Measured tumor size was determined by the formula [*x* (mm)^2^ * *y* (mm)]/2 = volume (mm^3^), where *x* refers to the shortest measurement and *y* the longest. Mice were sacrificed by cervical dislocation when the total tumor volume on either side was larger than 1,000 mm^3^. All experiments using animals were performed in accordance with the protocol approved by the Austrian Ministry (BMBWF-66.015/0009-V/3b/2019 or GZ: 340118/2017/25). Survival curves were plotted with the software GraphPad PRISM 8 (RRID:SCR_002798, v8).

### BLAST-Based Identification of HPV Genome Overlaps between Databases

A BLAST database was built from the reference fasta file using makeblastdb (BLAST+; ref. 41; v2.8.1), and query sequences were searched against this BLAST database with blastn (-outfmt 6 -max_target_seqs 1 -max_hsps 1). Query sequences with a sequence match ≥95% and sequence coverage ≥35% were considered as represented in the reference database.

### TCGA Reanalysis for HPV Associations

A custom-built Papillomavirus Episteme (PaVE, RRID:SCR_016599) database–based index for the Centrifuge metagenomics classifier (RRID:SCR_016665, v1.0.4) consisting of the human hg38 genome and 376 papillomavirus genomes (obtained from PaVE on August 24, 2018) was generated. All subsequent processing was performed using the Nextflow pipeline virus-detection-nf found under https://github.com/ObenaufLab/virus-detection-nf. Briefly, raw paired/unpaired TCGA tumor bam files (32 tumor types with a total of 10,078 tumor samples) were converted to fastq files using samtools (RRID:SCR_002105, v1.9) and analyzed using virus-detection-nf with Centrifuge (RRID:SCR_016665, v1.0.4). For each species, we calculated reads-per-million (RPM) and median absolute deviations as z-score across all samples. To classify a given sample as positive for a given virus, we require an RPM ≥5 and a corresponding *P* value of the z-score ≤0.05 for that specific virus.

### Identification of RF12 Gene Signature

TCGA-CESC and TCGA-HNSC expression data were downloaded from TCGA and annotated with HPV status based on our PaVE-based classification. Differentially expressed genes between HPV^+^ and HPV^−^ samples were independently called on raw counts of TCGA-CESC and TCGA-HNSC using DESeq2 (RRID:SCR_015687, v1.22.2). Genes with ≥10 counts and an adjusted *P* < 0.1 were considered differentially expressed and resulted in a gene set of 942 genes, which are commonly differentially expressed in TCGA-CESC and TCGA-HNSC with log_2_-fold changes in the same direction between HPV^+^ and HPV^−^ samples.

Expression data [transcripts per million (TPM)] of combined TCGA-CESC and TCGA-HNSC datasets were split into a training (60%) and a test (40%) set. Hyperparameters for an RF consisting of 1,000 trees were tuned, and a final RF with tuned parameters was fit to the training set using 10-fold cross-validation with the R packages tidymodels (https://www.tidymodels.org/, v0.1.4) and ranger (RRID:SCR_022521, v0.13.1). Importance scores for each gene were extracted, and the resulting gene list was filtered for protein-coding genes and ordered by decreasing importance. The top 12 genes covering a cumulative importance of 87% were picked as gene signature (RF12). Significantly enriched promoter motifs in RF12 signature genes were identified using Homer (RRID:SCR_010881, v4.11) with the findMotifs.pl script and default parameters. Enriched pathways in RF12 signature genes were identified using g:Profiler (RRID:SCR_018190) with default parameters. Cancer–testis genes were annotated based on a previous classification (67). RF12 gene expression in 76 cell types was obtained from the HPA project (68). Genes with a minimum expression level of 10 normalized transcripts per million (nTPM) were plotted. Cell type–specific enrichment analysis for RF12 genes was performed using PanglaoDB (RRID:SCR_022580). MHC-I antigens were predicted for RF12 genes, known cancer/testis antigens (MAGEA3, MAGEA4, MAGEA10, and NYESO1) and HPV16 and HPV42 oncogenes E6 and E7 using Net­MHCPan (RRID:SCR_018182, v4.1) with a peptide length of 9. Strong predicted binders were all peptides with a predicted %Rank <0.5 and weak predicted binders with a predicted %Rank <2.

### Machine Learning–Based Classification of HPV^+^ Tumor Samples

Expression data (TPM) of TCGA-CESC and TCGA-HNSC were combined with DPA expression data (CPM) and annotated with HPV status based on our PaVE-based classification. The resulting sample set was split into a training (60%) and a test (40%) set. For classification, we tuned hyperparameters for logistic regression models with glmnet (RRID:SCR_015505, v2.0–16), support vector machines with kernlab (ref. 69; v0.9-29), RFs with 1,000 trees using ranger (RRID:SCR_022521, v0.13.1), and single-layer neural networks with five hidden units using nnet (70; v7.3-12) in the tidymodels (https://www.tidymodels.org/, v0.1.4) framework. Each model was fitted with tuned parameters to the training set using 10-fold cross-validation. AUCs were extracted for each model on the test set, which yielded the random forest as the best-performing classifier for detecting HPV^+^ samples based on our RF12 gene signature. RF classification models based on CDKN2A, SYCP2, and RF2 (CDKN2A + SYCP2) were built as described above. GTEx normal tissue (obtained from the GTEx portal on May 11, 2021), HSIL (GSE150227), and normal skin/wart (GSE136347) expression data were classified with the indicated classification models.

### Data Availability

The datasets used in this study are available in the following databases: ViroCap DNA- and RNA-seq targeted sequencing data have been deposited in the Sequence Read Archive under accession number PRJNA812756. QuantSeq data of HPV-E7–transduced HPKs (GSE196217), HPV full-genome transduced HPKs (GSE196216), and DPA tumors (GSE196215) have been deposited in the Gene Expression Omnibus (GEO) database. The MS proteomics data have been deposited to the ProteomeXchange Consortium via the PRIDE partner repository with the accession number PXD031764 for quantitative TMT-MS and PXD031438 for proximity-labeling MS data. Public RNA-seq data used in this study are available from GEO under the accession numbers GSE136347 (skin wart and matched normal skin RNA-seq data), GSE150227 [normal, cervical intraepithelial neoplasia (CIN), and CESC RNA-seq data], and GSE121906 (noncanonical HIPPO signature). GTEx expression data were obtained from https://gtexportal.org/home/, TCGA expression data were obtained from https://gdc.cancer.gov/, and TCGA raw sequencing data were accessed via the database of Genotypes and Phenotypes (dbGaP) accession number phs000178.v11.p8.

## Supplementary Material

Supplementary Figures S1-S9 Supplementary data depicted in Supplementary Figures S1-S9

Supplementary Table S1 Sample-assay overview.

Supplementary Table S2 Viral abundance scores in ViroCap assay.

Supplementary Table S3 Viral genome coverage in ViroCap assay.

Supplementary Table S4 HPV42 integration sites in DPA.

Supplementary Table S5 FISH probes.

Supplementary Table S6 HPV42 protein-coding mutations.

Supplementary Table S7 HPV42 variant allele frequencies.

Supplementary Table S8 HPV42- and HPV16-E6 BioID.

Supplementary Table S9 HPV42- and HPV16-E7 BioID.

Supplementary Table S10 HPV abundance scores TCGA.

Supplementary Table S11 DEG HPV+ vs. HPV- CESC/HNSCC.

Supplementary Table S12 HPV42 consensus genomes.

Supplementary Table S13 HPV42 genome sequence comparison.

Supplementary Table S14 HPV42 genome structural variants.
