# Supplementary material for: Human Papillomavirus 42 Drives Digital Papillary Adenocarcinoma and Elicits a Germ Cell–like Program Conserved in HPV-Positive Cancers
Source: Cancer Discov. 2022 Oct 10;13(1):70–84. doi: 10.1158/2159-8290.CD-22-0489 (PMC9827110; doi:10.1158/2159-8290.CD-22-0489)
Supplement: Supplementary Figures S1-S9 — Supplementary data depicted in Supplementary Figures S1-S9 [file cd-22-0489_supplementary_figures_s1-s9_suppsf1.pdf]

Supplementary Figure S1

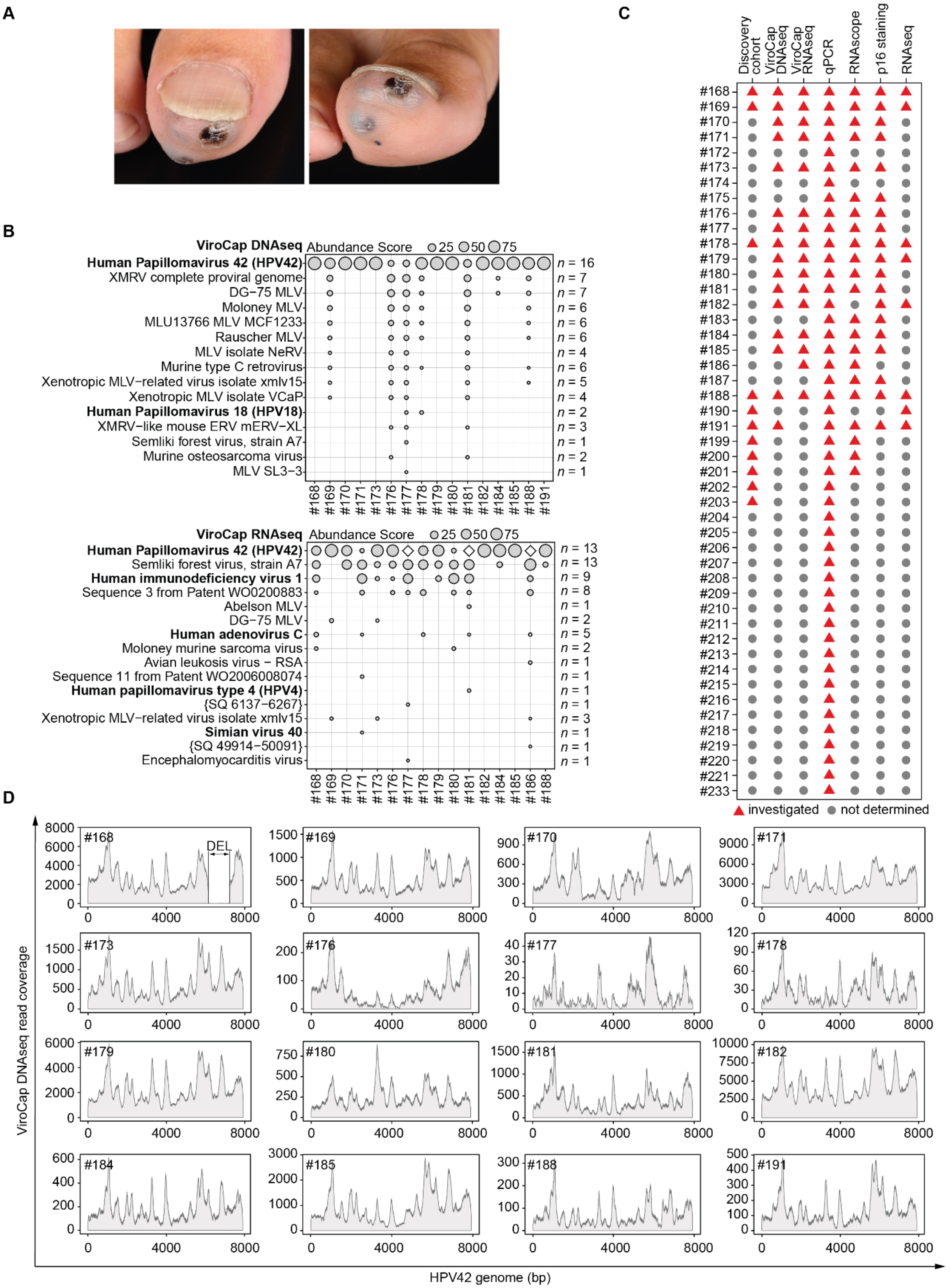

Supplementary Fig. S1: Detection of HPV42 in DPA. A, Clinical presentation of DPA. **B**, Abundance score of detected viral species in ViroCap DNA- and RNAseq. Viral species marked in bold infect humans as domestic hosts. White diamond: HPV42 detected but below an abundance of 1%. **C,** Overview of DPA sample-assay relations. Red triangle, sample investigated; gray dot, not determined **D**, HPV42 genome coverage plots in ViroCap DNAseq assay. Case #168 harbors a deletion (DEL) in the late region of the HPV42 genome.

Supplementary Figure S2

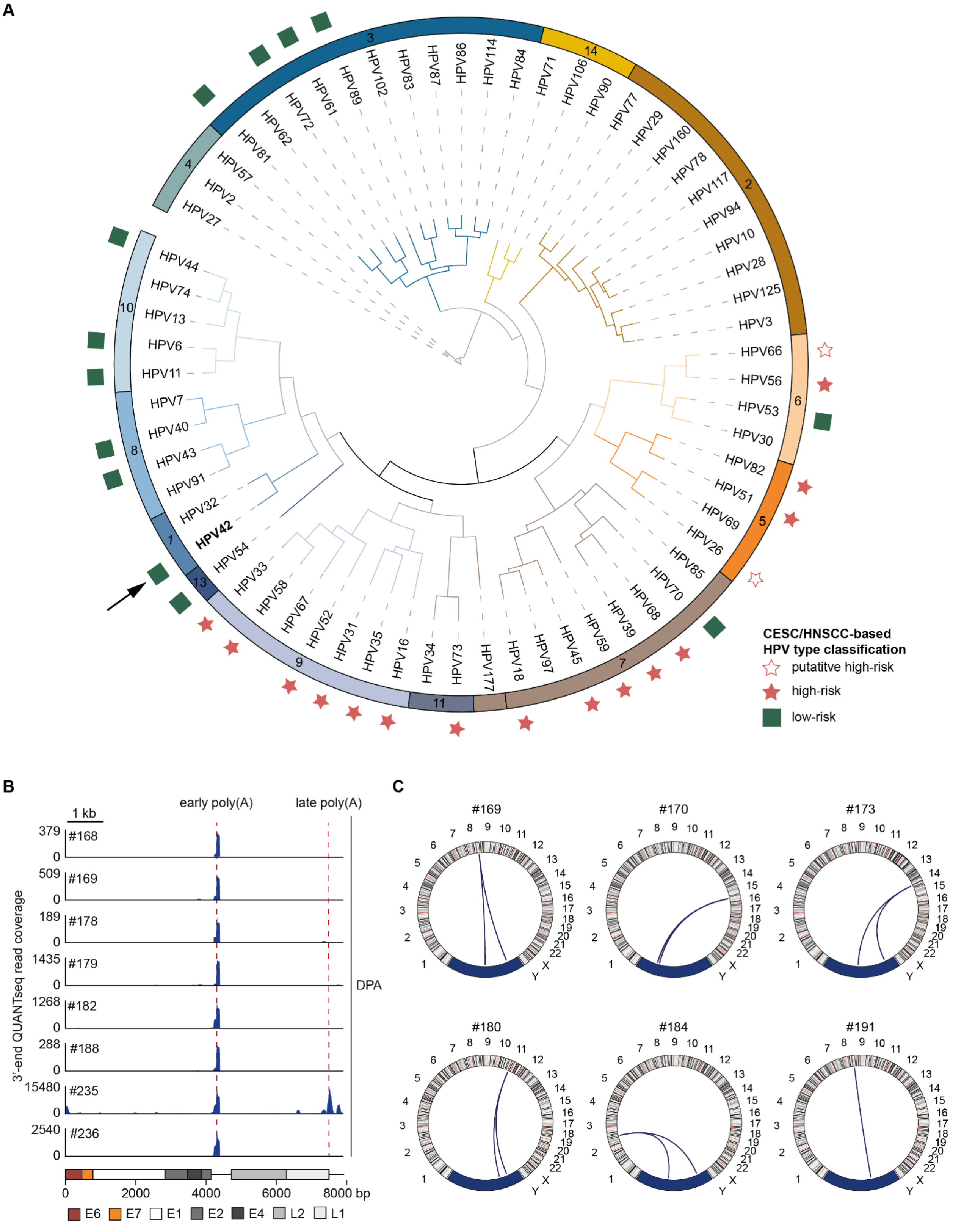

Supplementary Fig. S2: Phylogenetic and genomic characterization of HPV42 in DPA.

A, Phylogenetic tree of Alpha genus HPV types annotated with CESC/HNSCC-based HPV type classification. Green square, “low-risk” HPV types; red filled star, high-risk strains; red star with no filling, putative high-risk types. Black arrow, HPV42 genome. **B**, HPV42 gene expression profile in 3’-end RNAseq of 8 DPAs (early vs. late poly(A) signal). **C**, Circos plots of integration breakpoints between HPV42 and human genome. Each arch represents one integration breakpoint.

Supplementary Figure S3

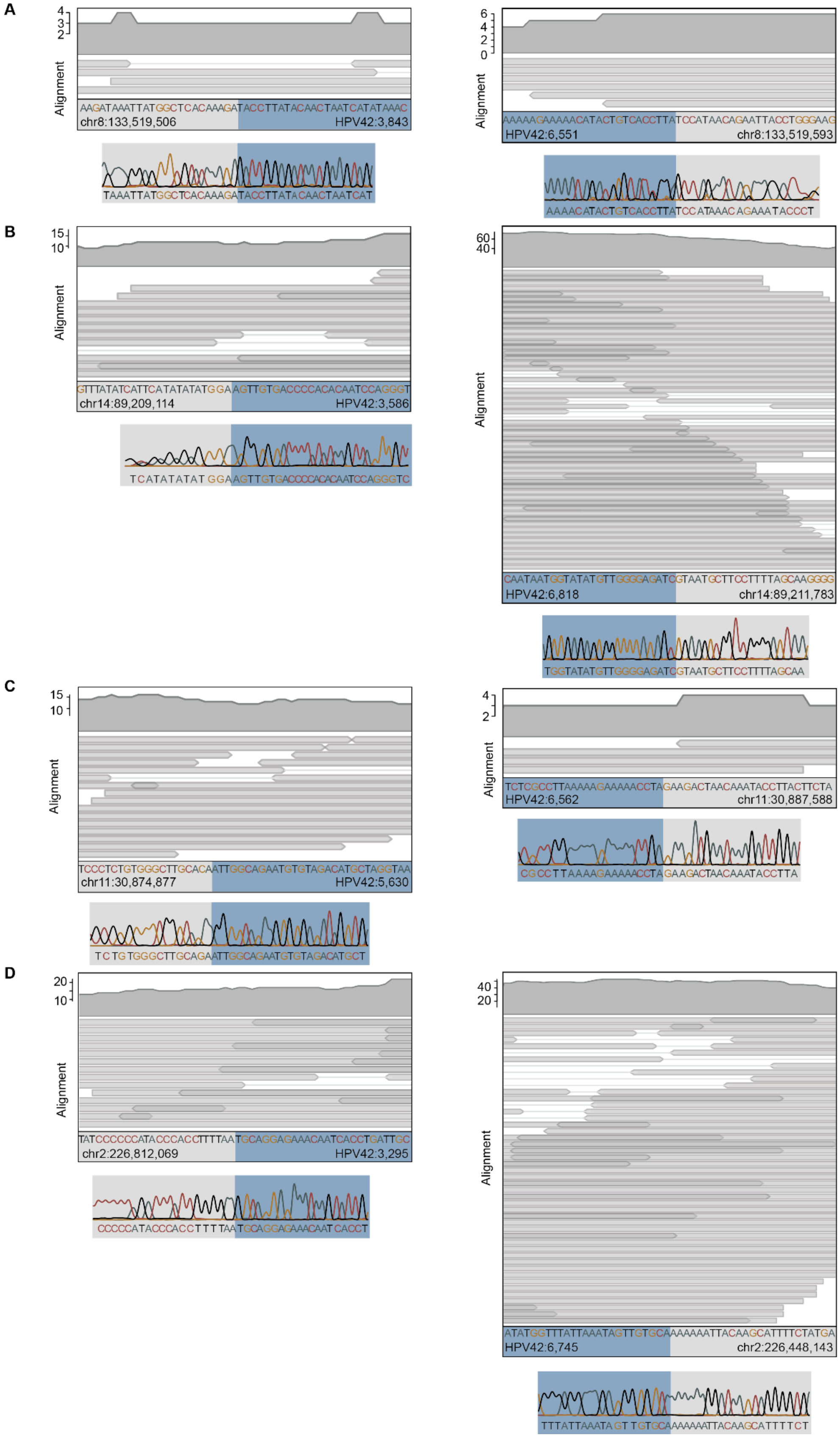

Supplementary Fig. S3: Representative HPV42 integration sites in DPA. Read alignments and Sanger sequencing tracks of HPV42-human genome breakpoints. For each breakpoint the base pair position in the respective reference genome HPV42 (PaVE) and human (hg38) is indicated. **A,** sample #169. **B,** sample #173, **C,** sample #180, **D,** sample #184.

Supplementary Figure S4

A

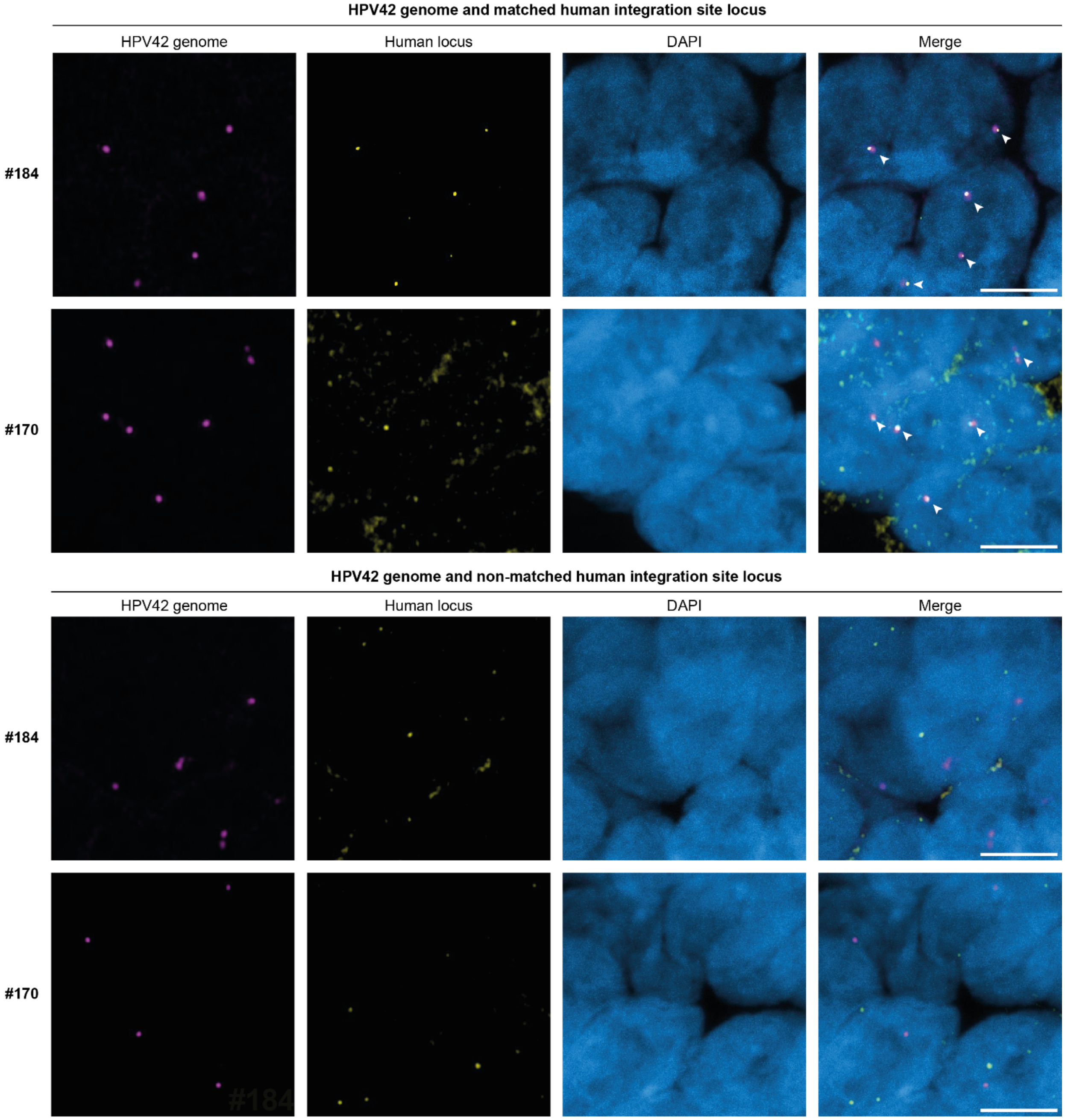

B

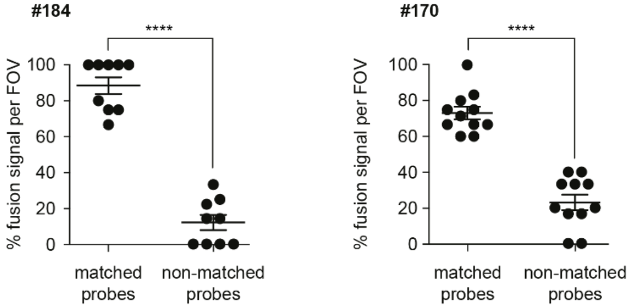

Supplementary Fig. S4: Fluorescence in situ hybridization (FISH) staining of HPV42 and integration site-adjacent human loci. **A**, (Top) Representative fields of view (FOV) showing HPV42 (magenta) and human (yellow) FISH staining signals for matched human integration site probes (#170.1 and #184.1) and (Bottom) non-matched human integration site probes (control) (Supplementary Table S4 and S5). White arrows indicate fusion signals. Scale bar 5µm. **B,** Quantification of nuclei with fusion signal between HPV42 and human loci. Each dot represents one quantified FOV in the respective condition. Horizontal line indicates the mean ± SEM. **** p <0.0001 (Two-tailed unpaired t test).

Supplementary Figure S5

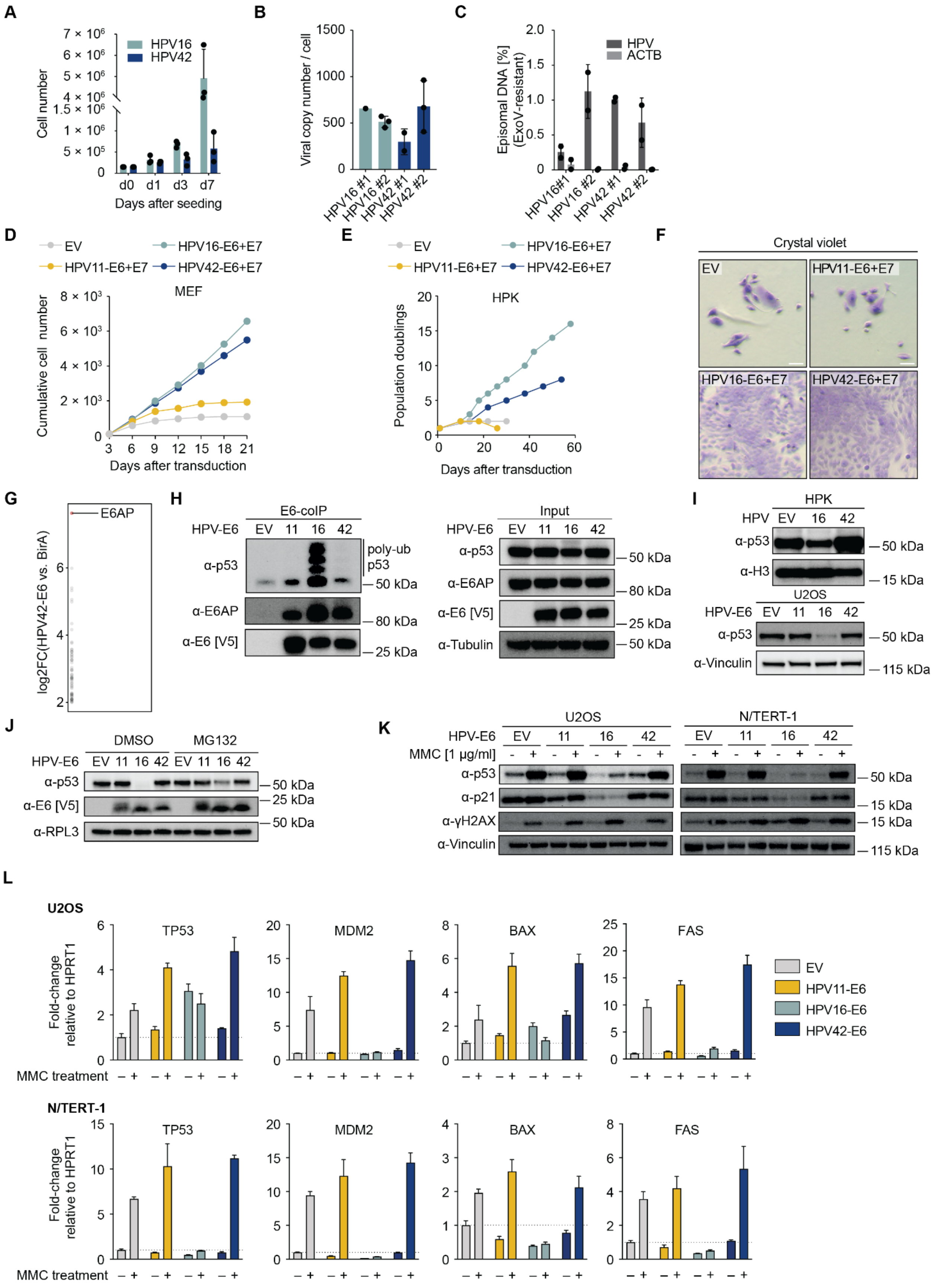

Supplementary Fig. S5: HPV42 extends the lifespan of primary cells. **A**, Short-term proliferation assay of HPK cells transduced with the full genome of HPV16 or 42. **B**, Viral copy numbers of HPK cells transduced with the full genome of HPV16 or 42. **C**, Fraction of episomal DNA in HPK cells transduced with the full genome of HPV16 or 42. **D**, Growth curves of MEF cells transduced with E6/E7 co-expression constructs of HPV11, 16, 42 or empty vector (EV). **E**, Growth curves of HPKs transduced with E6/E7 co-expression constructs of HPV11, 16, 42, or EV. **F**, Crystal violet staining of HPK cells at day 18 after transduction. Scale bar 100µm. **G**, HPV42-E6 protein-interaction partners identified in BioID proximity labeling experiment. E6AP/UBE3A labeled. **H**, Immunoblot of co-immunoprecipitation (coIP) of V5-tagged E6 proteins of HPV11, 16, and 42 in U2OS cells. **I**, (Top) Immunoblot of p53 in HPK cells transduced with the full genome of HPV16 or 42, or empty vector (EV). (Bottom) Immunoblot of p53 in U2OS cells, expressing wild-type p53, transduced with E6 proteins of HPV11, 16, 42, or EV. **J**, Immunoblot of p53 degradation assay in rabbit reticulocyte lysate. **K**, Immunoblot of p53, p21 and γH2AX in U2OS and N/TERT-1 cells following mitomycin c (MMC) treatment. **L**, RT-qPCR of TP53, MDM2, BAX and FAS in U2OS and N/TERT-1 cells following mitomycin c (MMC) treatment.

Supplementary Figure S6

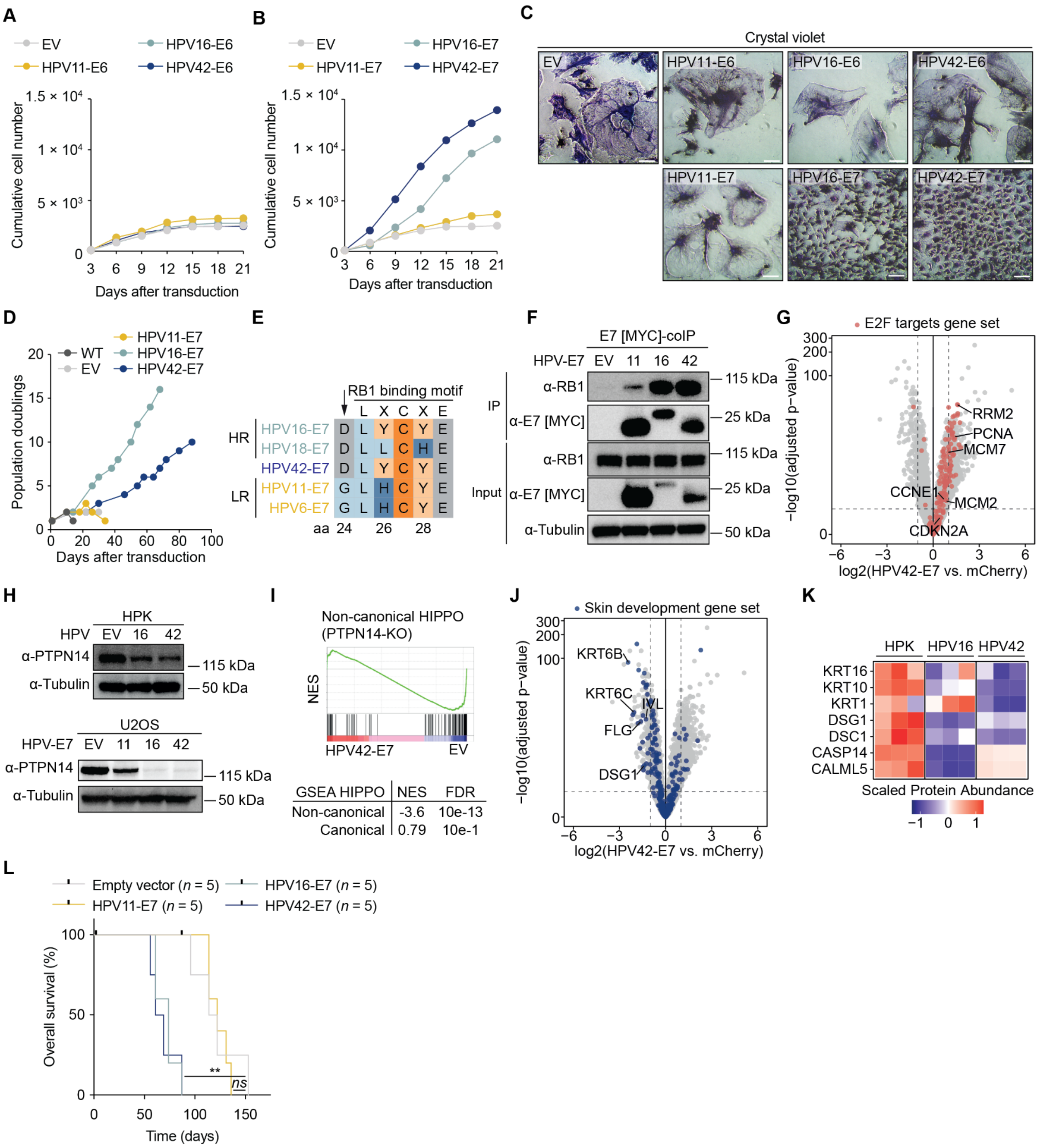

Supplementary Fig. S6: HPV42 shares cellular targets with high-risk HPVs. A, Growth curves of MEF cells transduced with E6 of HPV11, 16, 42, or EV. **B**, Growth curves of MEF cells transduced with E7 of HPV11, 16, 42, or EV. **C**, Crystal violet staining of MEF cells transduced with E6 (Top) or E7 (Bottom) of HPV11, 16, 42, or EV at day 21 after transduction. Scale bar 100μm. **D**, Growth curves of HPKs transduced with the E7 protein of HPV42, 11, 16, or EV. **E,** Sequence alignment of LxCxC RB1 binding motif of high-risk (HR) HPV16 and 18, low-risk (LR) HPV6 and 11, and HPV42. The conserved aspartic acid residue (D) -1 upstream of the LxCxC motif is indicated with an arrow. **F**, Immunoblot of co-immunoprecipitation (coIP) of MYC-tagged E7 proteins of HPV11, 16, and 42 in U2OS cells. **G**, Volcano plot showing the -log10(adjusted p-value) and log2 fold-change (log2FC) for transcripts detected by RNAseq comparing HPV42-E7 vs. empty vector (EV) transduced HPK cells. Members of the E2F target gene set are highlighted in red. Horizontal and vertical dashed lines indicate -log10(adjusted p-value) = 0.05 and log2FC = abs(1), respectively. **H**, (Top) Immunoblot of PTPN14 in HPK cells transduced with empty vector (EV), full genome of HPV16 or HPV42. (Bottom) Immunoblot of PTPN14 in U2OS cells transduced with E7 proteins of HPV11, 16, or 42. **I**, (Top) GSEA-enrichment of PTPN14-KO signature is significantly downregulated genes (-log10(adjusted p-value) $\leq$ 0.05 and log2FC $\leq$ -1) comparing HPV42-E7 vs. empty vector (EV) transduced HPK cells. (Bottom) GSEA-enrichment of canonical and non-canonical HIPPO signaling gene sets comparing HPV42-E7 vs. empty vector (EV) transduced HPK cells (Bottom). NES, normalized enrichment score. **J**, Volcano plot showing the -log10(adjusted p-value) and log2 fold-change (log2FC) for transcripts detected by RNAseq comparing HPV42-E7 vs. empty vector (EV) transduced HPK cells. Members of the skin development gene set are highlighted in blue. Horizontal and vertical dashed line indicate -log10(adjusted p-value) = 0.05 and log2FC =

abs(1), respectively. **K**, Quantitative TMT-mass spectrometry data for genes included in GO-term "skin differentiation" of HPK cells transduced with the full genome of HPV16 or 42, or EV. **L**, Kaplan-Meier curve of EV, or E7 of HPV11 ($p = 0.8719$), HPV16 ($p = 0.0019$) or HPV42 ($p = 0.0027$) transduced HaCaT cell tumors. Mice were sacrificed when tumors reached a total volume of $\geq 1cm^3$. ** $p < 0.01$; ns, non-significant (log-rank Mantel-Cox test).

Supplementary Figure S7

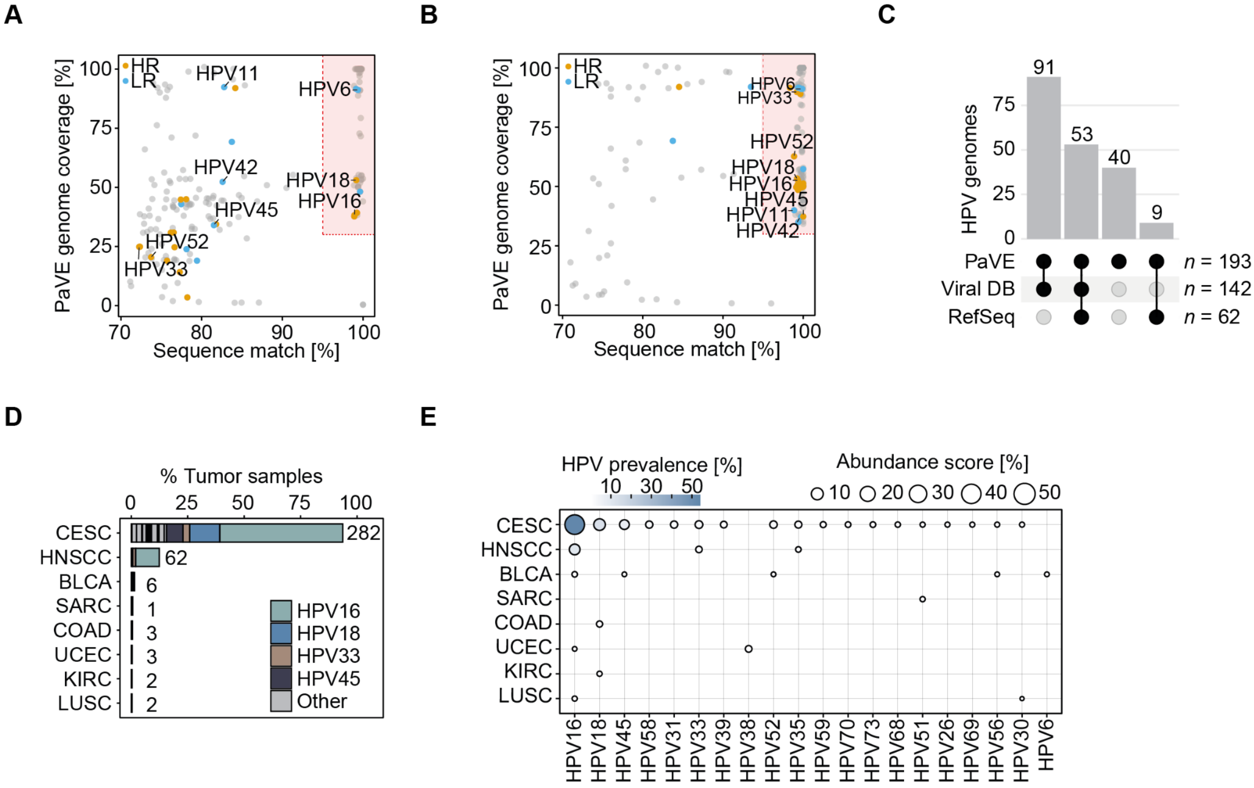

Supplementary Fig. S7: The NCBI RefSeq database does not include most HPV genomes and HPV42 is not found in common cancer types. A, Nucleotide BLAST (BLASTN) search of PaVE database HPV genomes against NCBI RefSeq database. HR, high-risk HPV; LR, low-risk HPV. Each dot represents one HPV genome. HPV genomes with a sequence match ≥ 95% and a genome coverage ≥ 35% shared between databases are considered matching. **B**, Nucleotide BLAST (BLASTN) search of PaVE database HPV genomes against Viral DB. HR, high-risk HPV; LR, low-risk HPV. Each dot represents one HPV genome. HPV genomes with a sequence match ≥ 95% and a genome coverage ≥ 35% shared between databases are considered matching. **C**, Upset plot illustrating the overlap of HPV genomes between Papillomavirus Episteme reference database (PaVE), Viral DB, and NCBI RefSeq database. Viral DB is the custom vertebrate virus genome database used in Fig. 1A. **D**, HPV prevalence [%] across all tumors in TCGA (n = 10,087, 32 cancer types), assessed using the PaVE database. CESC, Cervical Squamous Cell Carcinoma, and Endocervical Adenocarcinoma; HNSCC, Head, and Neck Squamous Cell Carcinoma; COAD, Colon Adenocarcinoma; UCEC, Uterine Corpus Endometrial Carcinoma; SARC, Sarcoma; BLCA, Bladder Urothelial Carcinoma; KIRC, Kidney Renal Clear Cell Carcinoma; LUSC, Lung Squamous Cell Carcinoma. **E**, Abundance score of all HPV types (n = 19) detected in TCGA tumors (n = 8 tumor types). Viral prevalence: Proportion of samples with detected virus in any given cancer type. Abundance score: Mean proportion of detected virus in any given cancer type.

Supplementary Figure S8

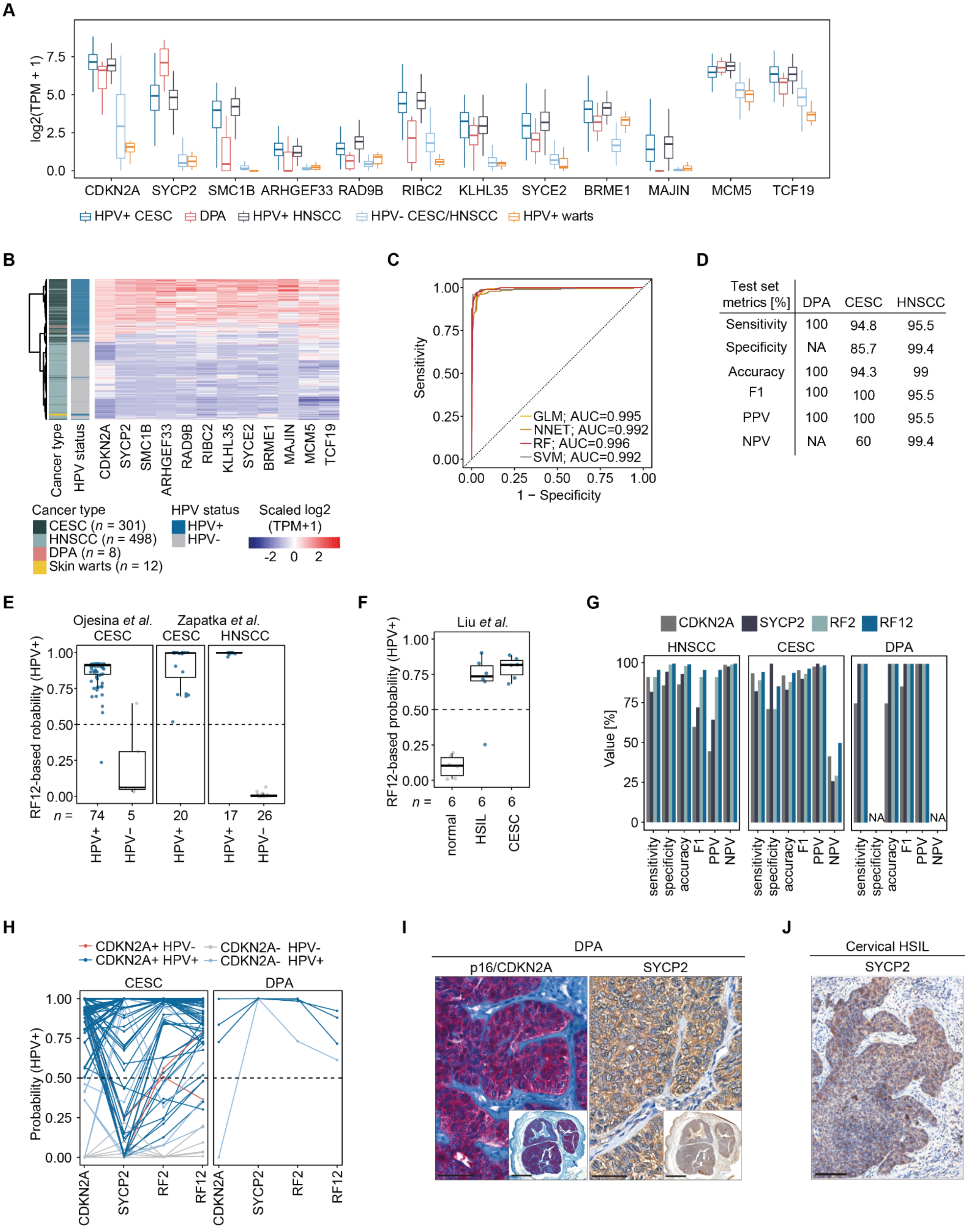

Test set metrics [%]	DPA	CESC	HNSCC
Sensitivity	100	94.8	95.5
Specificity	NA	85.7	99.4
Accuracy	100	94.3	99
F1	100	100	95.5
PPV	100	100	95.5
NPV	NA	60	99.4

Supplementary Fig. S8: HPV-driven tumors share a conserved transcriptional signature. A, Expression levels of RF12 signature genes in DPA, CESC, HNSCC, and skin warts. CESC and HNSCC samples are stratified by HPV status assessed using our PaVE-based annotation. **B**, Heatmap of RF12 signature gene expression levels in CESC, HNSCC, DPA, and warts. **C**, Receiver operating characteristic (ROC) curves with corresponding area under the curve (AUC) values for the generalized linear model (GLM), neuronal network (NNET), random forest (RF), and support vector machine (SVM) machine learning approaches in the testing set across CESC, HNSCC, and DPA. **D**, Performance metrics of RF12 illustrated by sensitivity, specificity, accuracy, F1 score, positive predictive value (PPV), and negative predictive value (NPV). **E**, HPV+ probability scores calculated by RF12 for CESC and HNSCC samples from Ojesina *et al.* and Zapatka *et al.* **F**, HPV+ probability scores calculated by RF12 for normal, CIN2/3 and CESC samples from Lui *et al.* **G**, Performance metrics of CDKN2A and SYCP2 alone, in combination (RF2) or RF12 illustrated by sensitivity, specificity, accuracy, F1 score, positive predictive value (PPV), and negative predictive value (NPV). **H**, HPV+ probability scores for CESC and DPA calculated for CDKN2A and SYCP2 alone, the combination of CDKN2A and SYCP2 (RF2) and RF12. **I**, IHC of p16/CDKN2A (left) and SYCP2 (right) in DPA. Scale bar, main: 50µm; insert: 3mm. **J**, IHC of SYCP2 in CESC. Scale bar, main: 100µm.

Supplementary Figure S9

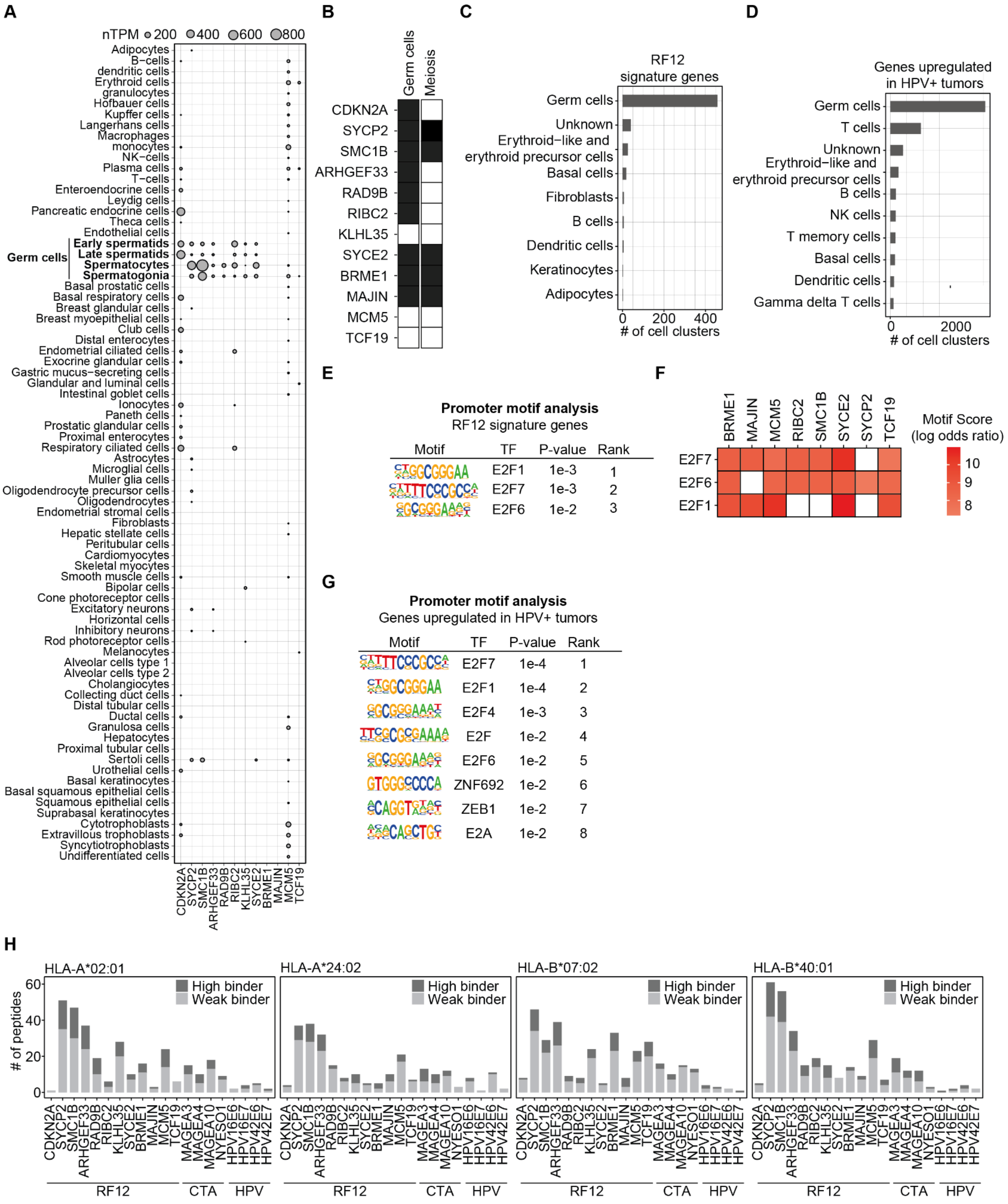

Supplementary Fig. S9: HPV-driven tumors share a germ cell-like transcriptional program. A, Transcript expression levels for RF12 genes in 76 cell types. nTPM, normalized transcripts per million. **B**, RF12 annotation for genes involved in meiotic cell cycle regulation. **C**, Cell-type specific expression enrichment analysis for RF12 signature genes. **D**, Cell-type specific expression enrichment analysis of protein-coding genes upregulated in HPV+ tumors. **E**, Promoter motif analysis of RF12 signature genes. **F**, E2F-family promoter motifs enriching in RF12 signature genes. **G**, Promoter motif analysis of protein-coding genes upregulated in HPV+ tumors. **H**, MHC-I antigen peptide predictions for RF12 signature genes (RF12), known cancer/testis antigens (CTA) and HPV16 and -42 oncogenes (HPV).
